# Microneedle technology in the treatment of ocular diseases: Advances and applications

**DOI:** 10.1016/j.ajps.2026.101129

**Published:** 2026-01-31

**Authors:** Jing Li, Zi Yan, Dengxuan Mao, Xiumei Liu, Peixin Lee, Yaqi Lyu, Nianping Feng

**Affiliations:** School of Pharmacy, Shanghai University of Traditional Chinese Medicine, Shanghai 201203, China

**Keywords:** Microneedle technology, Drug delivery, Anterior segment diseases, Posterior segment diseases, Ocular physiological barriers

## Abstract

Microneedle (MN) technology has emerged as a transformative approach for treating ocular diseases, addressing the limitations of conventional drug delivery methods such as low bioavailability and poor patient compliance. This review highlights the advancements and applications of MNs in managing both anterior and posterior segment ocular diseases. MNs overcome physiological barriers, enabling precise, minimally invasive drug delivery with enhanced efficacy. Various MN designs, such as solid, coated, soluble, hollow, hydrogel and cryo-MNs, are tailored for specific therapeutic needs, offering rapid or sustained drug release. Applications include treating keratitis, glaucoma, age-related macular degeneration and diabetic retinopathy, demonstrating improved drug penetration and reduced side effects. Despite challenges in manufacturing and clinical translation, MN technology holds promise for revolutionizing ocular therapeutics through innovations in materials, design, and personalized medicine.

## Graphical abstract

**Figure:**
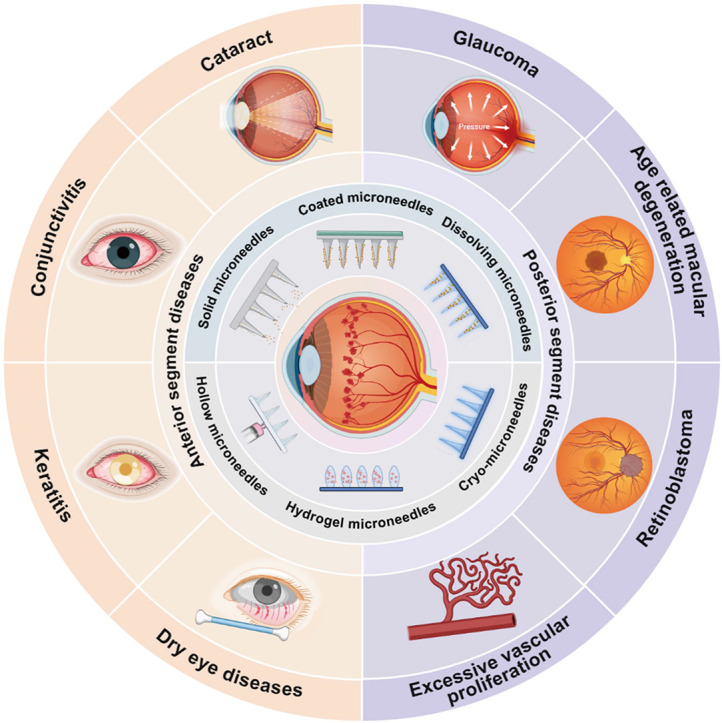

## 1 Introduction

Ocular diseases represent a leading cause of visual impairment and blindness worldwide, with both incidence and disease burden increasing annually. According to the World Health Organization (WHO), approximately 2.2 billion individuals are affected by visual impairment, with at least 1 billion of these cases considered either preventable or inadequately treated [1]. Ocular diseases are primarily classified into two categories: anterior segment disorders, including cataracts and glaucoma, and posterior segment pathologies, such as age-related macular degeneration (AMD) and diabetic retinopathy (DR). Cataracts remain the world’s leading cause of blindness, impacting over 94 million people [2]. Glaucoma ranks as the second leading cause, which is projected to affect 110 million by 2040 [3]. In developed countries, AMD is the primary cause of severe vision loss among the elderly, and the number of affected individuals is expected to increase to 288 million by 2040 [4]. Furthermore, the prevalence of DR is also rising steadily, paralleling the increasing incidence of diabetes mellitus. Additionally, many ocular diseases are strongly associated with aging, suggesting a continued escalation in the burden of ocular disease [5].

Current treatments for ocular diseases mainly include medication, laser therapy, and surgical interventions. Among these, topical and systemic medications are the most widely used due to their non-invasiveness and accessibility [6]. Common pharmaceutical formulations include eye drops, ointments, gels, and intraocular injections, each of which has its own unique advantages and limitations [7]. Eye drops are the most frequently administered dosage form because of their ease of use and rapid onset of action. They are extensively employed in the treatment of glaucoma, dry eye diseases (DED) and ocular infections. According to research, the global eye drop market was valued at USD 15.79 billion in 2022 and is projected to grow at a compound annual growth rate (CAGR) of 5.3%, reaching USD 23.79 billion by 2030. Despite their widespread application and convenience, anatomical and physiological ocular barriers, including tear turnover and corneal permeability, severely restrict their bioavailability, with typically less than 5% of the administered drug reaching target tissues. Furthermore, the requirement for frequent administration (e.g., 2–4 times daily for glaucoma) often leads to poor patient adherence, undermining therapeutic outcomes [8]. Eye ointments and gels are typically used in conditions requiring continuous treatment, such as nighttime therapy or corneal repair, because of their sustained drug release properties. These formulations create a protective film on the ocular surface, thereby significantly prolonging the action duration and reducing the administration frequency.

**Fig. 1:**
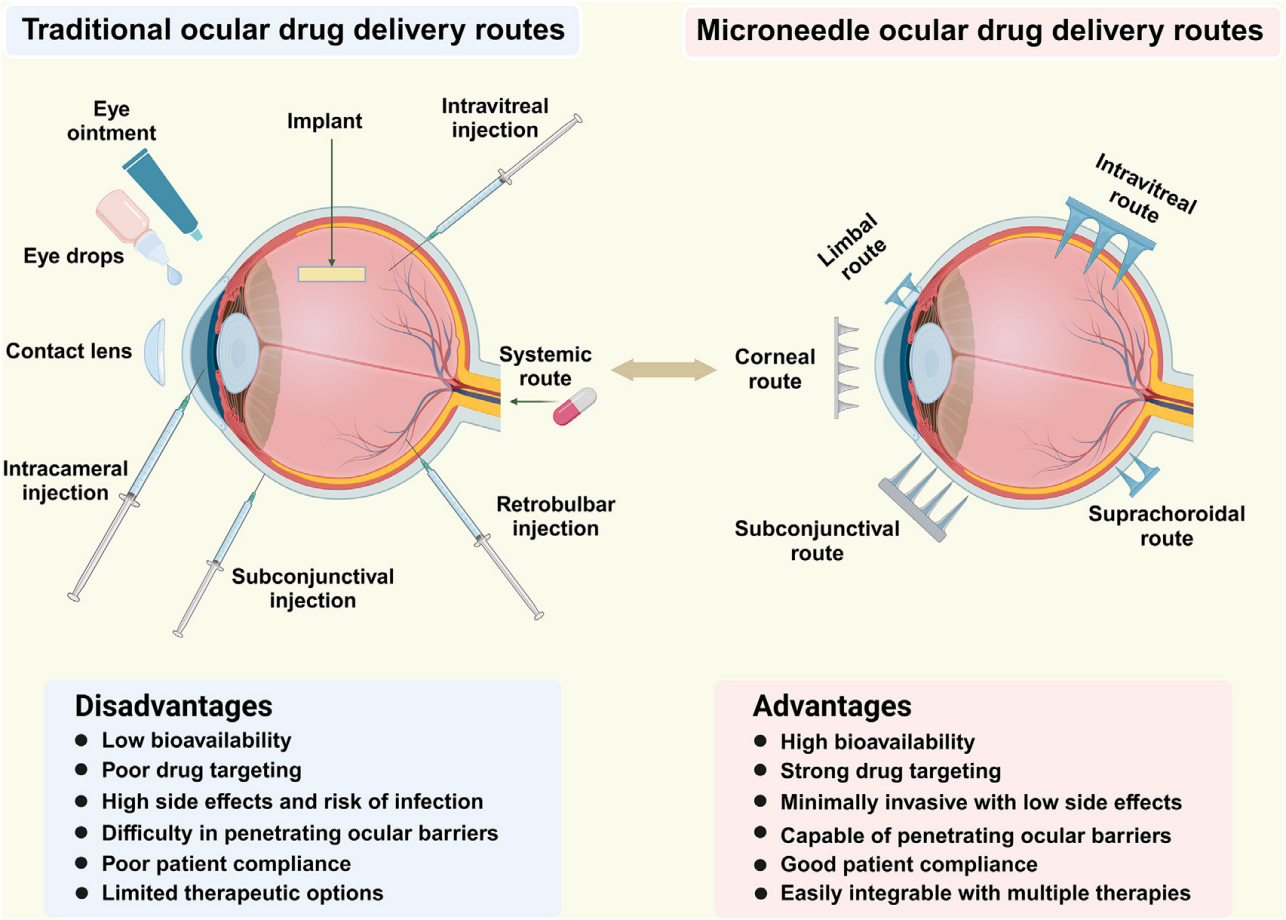
Comparison between traditional and microneedle-based ocular drug delivery. (Created with BioRender.com).

For example, certain antibiotic eye ointments require application only once or twice daily. However, the viscous nature of these formulations often causes blurred vision and ocular discomfort after application, which may reduce patient compliance. Intraocular injections have become a key treatment of various retinal diseases (RD) such as AMD, diabetic macular edema, and retinal vein occlusion (RVO). By delivering therapeutics directly into the vitreous cavity, they achieve high local drug concentrations and minimize systemic exposure [9]. Market data indicate that the global ocular injection market will reach a value of USD 11.98 billion in 2023, and is expected to grow to USD 21.2 billion by 2032, with a CAGR of 6.54%. Nevertheless, intravitreal injections carry risks of endophthalmitis, cataract genesis, and elevated intraocular pressure (IOP) [10]. Systemic administration via oral or intravenous routes is occasionally used as an adjunctive approach for infectious uveitis, endophthalmitis, or immune-mediated ocular conditions [11]. However, the blood-retinal barriers (BRB) significantly limit intraocular drug penetration, often resulting in bioavailability below 2% [12]. This necessitates high systemic doses to achieve therapeutic drug concentrations within the eye, leading to high risks of adverse effects such as gastrointestinal, hepatic, or renal toxicity. In summary, conventional ocular drug delivery systems are constrained by poor bioavailability, inadequate patient compliance, limited targeting precision, and potential local and systemic side effects, thus more efficient and safer ocular drug delivery systems are urgently needed to address these challenges (Fig. 1).

MNs, as an innovative drug delivery system, have undergone rapid advancement since their invention in the late 1990s [13]. The core principle of MNs involves penetrating the stratum corneum of the skin using micrometer-sized needle-like structures to deliver drugs with minimal pain and invasiveness [14]. As the fabrication technology and materials science have advanced, the applications of MNs have progressively expanded beyond ocular, oral, and transdermal drug delivery to include vaccine administration, cancer therapy, and chronic disease management. In fact, several MN-based systems are currently undergoing clinical trials [15,16]. The unique advantages of MN technology lie in its ability to overcome significant limitations associated with traditional drug delivery, thus offering more efficient and precise treatments.

Currently, numerous challenges exist in the treatment of ocular diseases, primarily due to physiological barriers within the eye. These protective barriers severely limit the effectiveness of conventional ocular drug delivery. MNs exhibit substantial advantages and promising application potential for ocular drug delivery, as they can efficiently penetrate ocular barriers, including the corneal epithelium, enabling direct delivery of therapeutic agents to target ocular tissues such as the corneal stroma, anterior chamber, vitreous body, retina, or choroid. By precisely targeting ocular tissues with micro-sized needles, MNs overcome the low bioavailability often seen with conventional formulations like eye drops and ointments. Their minimally invasive and painless nature enhances patient experience, minimizes the risk of complications with traditional intraocular injections such as infection and bleeding, consequently improving patient compliance. Moreover, MNs exhibit strong adaptability. MN materials and geometric parameters can be customized to achieve either rapid or sustained drug release, thereby meeting the diverse therapeutic demands in clinical practice. Collectively, these benefits establish MNs as a highly promising platform for ocular drug delivery, offering innovative therapeutic solutions for a range of ocular diseases. Fig. 1 highlights the limitations of traditional ocular drug delivery methods and the advantages of MN technology.

**Fig. 2:**
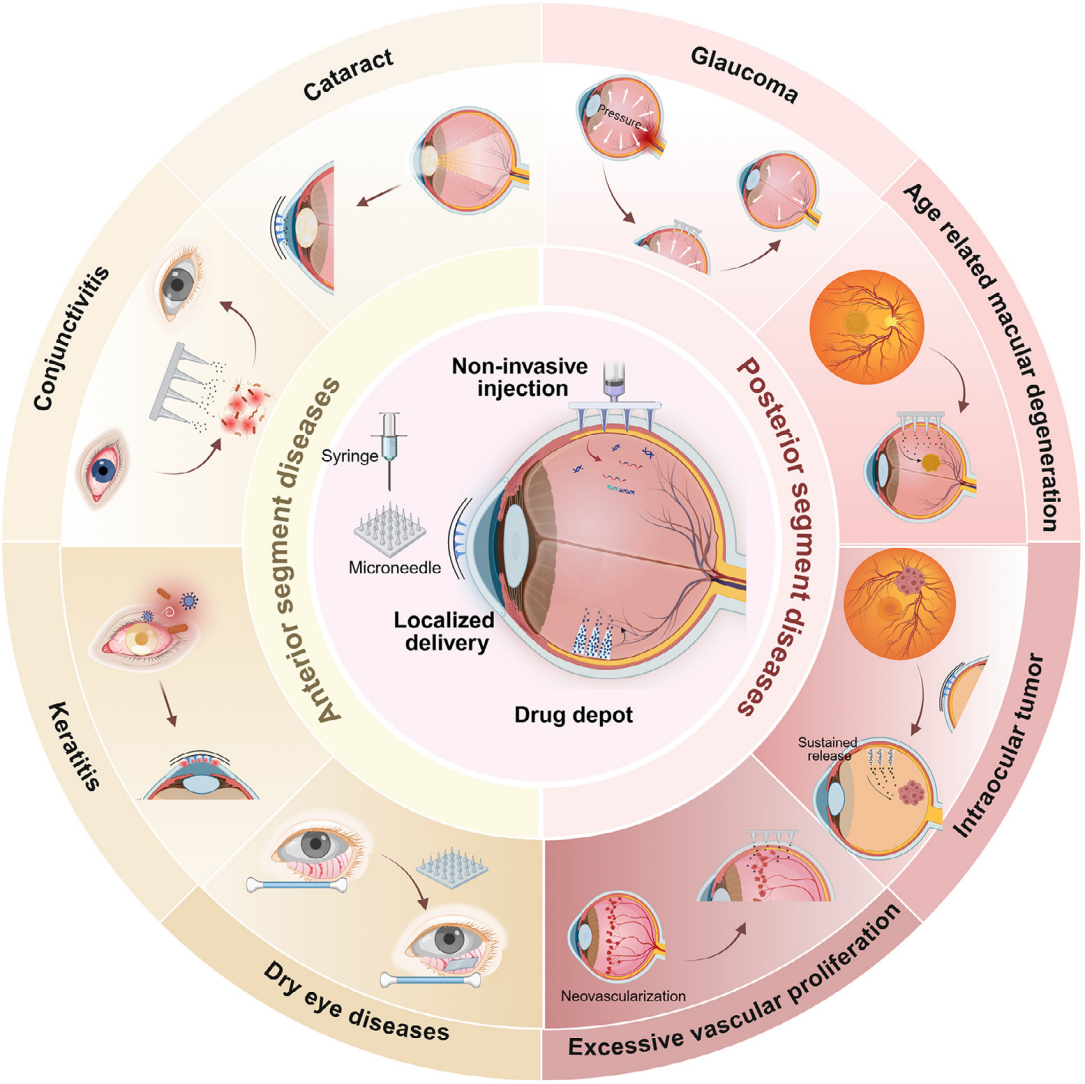
Schematic illustration of MN applications in both anterior and posterior ocular diseases (Created with BioRender.com).

This review systematically summarizes existing advances of MN techniques, as well as their potential applications, and future directions in treating various ocular diseases (Fig. 2). Through a detailed analysis of the unique strengths of MNs, including efficient penetration of ocular barriers, precise targeted drug delivery, and minimally invasive, nearly painless administration, we discuss their promising applications in treating diseases of both the anterior and posterior segments of the eye. This review aims to stimulate further innovation in MN technology and facilitate the development of safer, more effective therapeutic strategies for ocular disorders.

## 2 MN technology

### 2.1 Design and classification of MNs

**Fig. 3:**
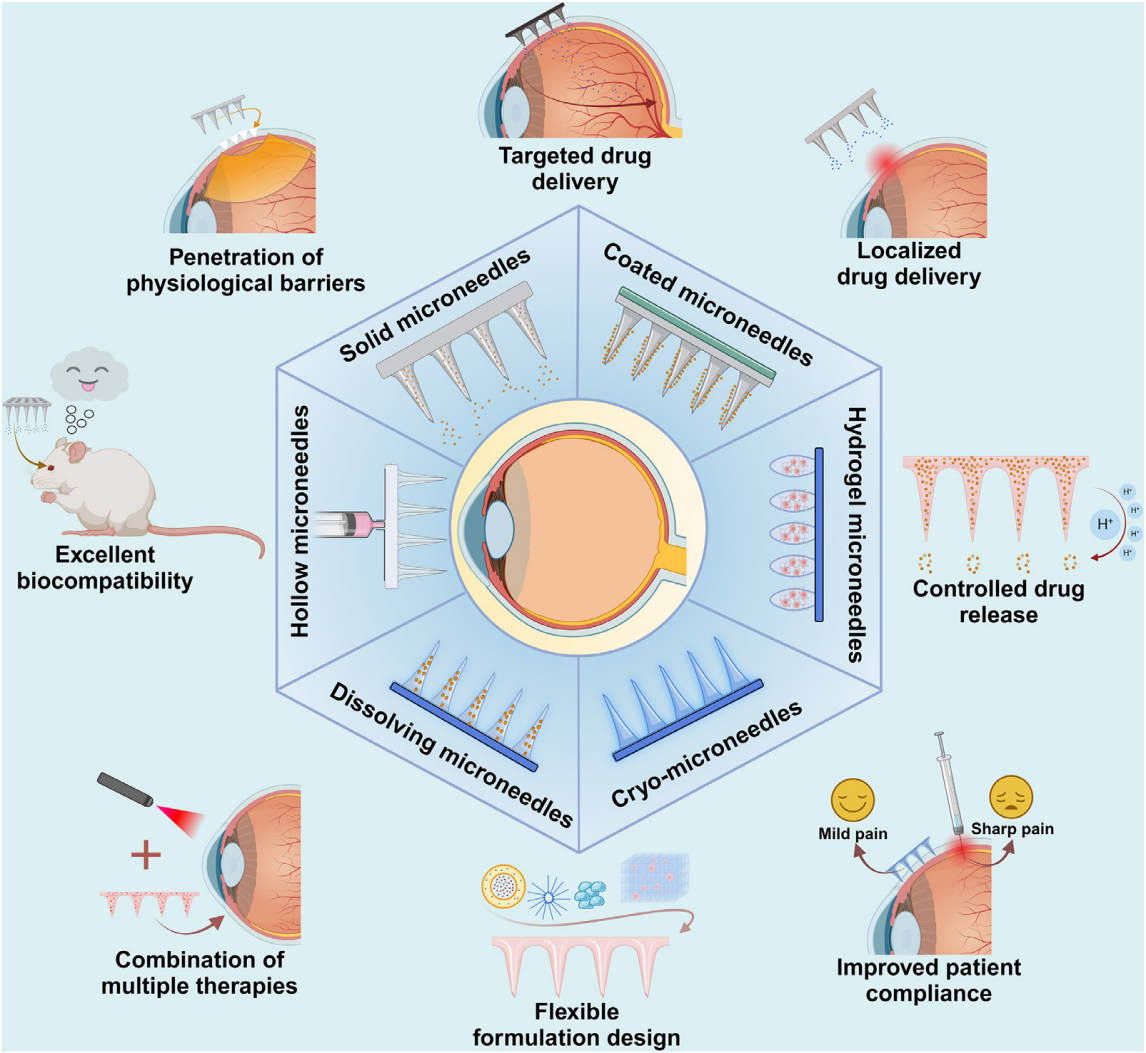
Six types of MNs and their applications in ocular drug delivery. (Created with BioRender.com).

The physical and chemical properties of MNs are core factors in determining their performance and application range, directly affecting their biocompatibility, mechanical properties and drug release. Table 1 summarizes the characteristics of various types of MNs. In accordance with the nature of materials and application requirements, MNs can be categorized as solid MNs, coated MNs, soluble or dissolving MNs, hollow MNs, hydrogel MNs and cryo MNs. Upon application of these MNs in the eye, they can achieve various functions such as localized drug delivery, targeted drug delivery, controlled drug release, etc. Fig. 3 illustrates a schematic diagram of major functions that these 6 types of MNs can achieve in ocular application.

**Table 1:** Preparation methods and characteristics of different types of MNs.

Type	Manufacturing technique	Commonly used materials	Advantages	Disadvantages	Type of drug suitable for delivery
Solid MNs	Photolithography and electrochemical etching	Metals or high-strength polymers	High mechanical strength, simple to use	May cause tissue damage and immune response, risk of infection	Hydrophobic drugs, small-molecule drugs and vaccines
Coated MNs	Dip or spray coating	Large polymers such as PVA and PEG	High drug loading, easy to handle	Uneven coating, difficult to control the release rate	Rapid release drugs such as local anesthetics and vaccines
Soluble MNs	Molding method	Water-soluble macromolecules such as HA, polysaccharides, and PVP	Biocompatible, no removal, lower side effects	Longer drying time	Hydrophilic drugs, macromolecular biopharmaceuticals, and vaccines
Hollow MNs	Photolithography and micromolding	Metals or high-strength polymers	Rapid drug release	Rapid drug release, complex manufacturing processes, higher costs, tip clogging	Liquid drugs, high-dose drugs, and large molecules
Hydrogel MNs	Photopolymerization and 3D printing	Polymers or composites, etc.	Relatively short preparation time, slow-release properties, reduced frequency of drug delivery	Low mechanical strength, difficult to control drug release	Hydrophilic drugs, macromolecular biopharmaceuticals, and smart-responsive drugs
Cryo-MNs	Rapid freezing method	Glycerol and polyethylene glycol, etc.	Good formability at low temperatures, rapid melting at physiological temperatures	Strictly controlled cryogenic conditions for preparation and storage	Heat-sensitive drugs, living cells, and biomolecules

#### 2.1.1 Solid MNs

**Fig. 4:**
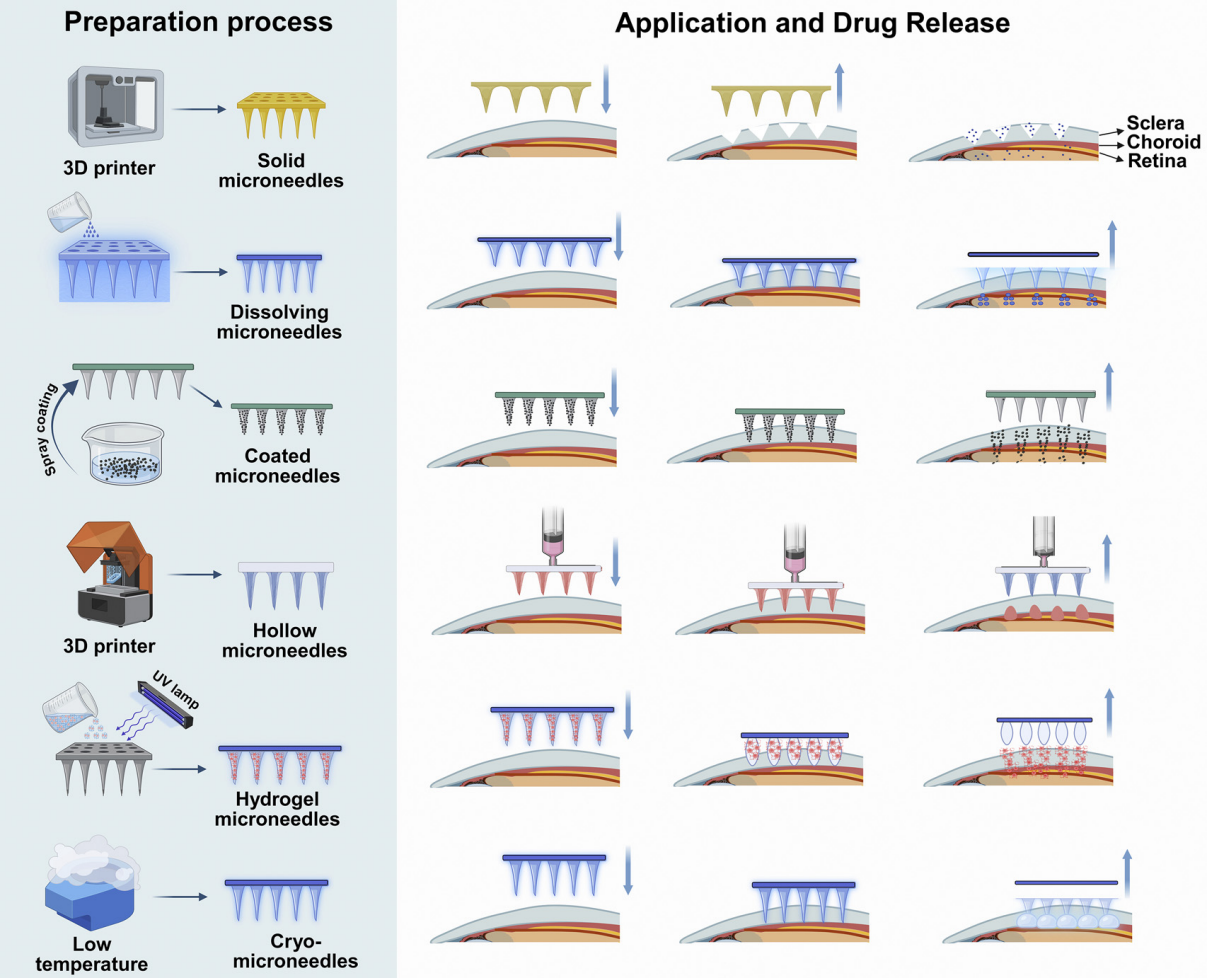
Illustrations of fabrication methods and drug delivery mechanisms of six types of MNs. (Created with BioRender.com).

Solid MNs are predominantly fabricated from metals (e.g., stainless steel, titanium alloys) or robust and nondegradable polymers (e.g., polycarbonate), providing the excellent mechanical properties necessary for deep tissue penetration in demanding therapeutic applications [17]. These devices function by mechanically generating transient microchannels within tissues. Upon MN removal, administered drugs diffuse through these pathways to reach the targeted sites. The primary fabrication techniques include photolithography and electrochemical etching. Photolithography defines MN patterns on silicon wafers utilizing photomasks and photoresists prior to metal electrodeposition, whereas electrochemical etching directly forms MN arrays on metal surfaces via controlled electrochemical reactions [18]. Fig. 4 illustrates the preparation methods of various kinds of MNs and their drug delivery process upon application in the eye. In ocular therapy, solid MNs effectively penetrate the corneal epithelium, creating temporary microchannels that facilitate localized drug administration, thereby enhancing anti-angiogenic efficacy in treating conditions such as corneal neovascularization (CNV) [19]. However, the nondegradable nature of solid MNs poses clinical concerns, including potential tissue injury, immune reactions, the need for device removal, and heightened risks of infection.

#### 2.1.2 Coated MNs

Coated MNs deliver therapeutic agents via surface coatings that rapidly dissolve upon tissue penetration, enabling immediate drug release into targeted regions. This design facilitates rapid pharmacological action, making it particularly advantageous for therapies that require a swift onset. The drug loading capacity is governed primarily by the geometry of MN tips. Common manufacturing methods include dip-coating and spray-coating techniques. Dip-coating achieves uniform drug deposition by precisely controlling immersion time and solution concentration [20], whereas spray-coating ensures accurate and uniform drug loading through controlled deposition of solutions. The coatings typically consist of biocompatible, rapidly dissolvable polymers such as polyvinyl alcohol (PVA) and polyethylene glycol (PEG) [21]. In ocular therapeutics, coated MNs demonstrate significant potential. For instance, in managing CNV, immediate anti-angiogenic drug release upon corneal penetration facilitates drug diffusion, thus effectively inhibiting the formation of pathological vessels.

#### 2.1.3 Soluble MNs

Soluble MNs are typically made by embedding therapeutic agents into water-soluble matrices such as hyaluronic acid (HA) or gelatin. Upon insertion into tissue, the MNs dissolve rapidly, releasing the drug directly at the site. When constructed from biodegradable and biocompatible polymers, these MNs eliminate the need for post-application removal, reducing the risk of surgery-induced complications. For example, Zhang et al. [22] designed a bilayer soluble MN system for treating atopic dermatitis. The tips release antioxidant nanoparticles to scavenge reactive oxygen species (ROS), while the base delivers probiotics that remodel the skin microbiome. This synergistic action simultaneously reduces oxidative stress and restores microbial balance, leading to improved therapeutic outcomes. Soluble MNs are usually made by casting polymer mixtures into a mold under centrifugal or vacuum conditions, then solidifying them through drying or UV curing. The excellent biocompatibility and safety profile of soluble MNs make them well suited for ocular use. For instance, in treating keratoconus, soluble MNs create transient microchannels on the corneal epithelium surface, increasing drug penetration through the cornea. During the process of MN dissolution, the drug is released in a sustained manner to reduce corneal refractive errors, thereby improving visual outcomes [23]. Similarly, scleral-penetrating soluble MNs directly deliver drugs to the posterior segment via sclera puncture, resulting in sufficient intraocular drug concentrations for improved treatment effectiveness [24].

#### 2.1.4 Hollow MNs

Hollow MNs function as miniature syringes. They feature a tiny opening at the tip that allows drug solutions or dispersions to be injected directly into target tissues after insertion. Drug delivery by hollow MNs can be achieved not only via passive diffusion driven by tissue fluid concentration gradients, but also through active administration using applied pressure, such as micropumps. This design is particularly suitable for the precise delivery of large-dose medications [25]. Hollow MNs are typically fabricated via photolithography and micromolding techniques, using materials such as silicon and polycarbonate. Owing to their controllable operation and high delivery precision, hollow MNs have already been used clinically to treat retina-related diseases [26]. In the treatment of glaucoma, drugs can be injected directly into the ciliary body to reduce IOP, reflecting the targeted delivery advantages [27]. Despite this, the clinical translation of hollow MNs still faces some hurdles. The geometric complexity of the needle tips makes the manufacturing process complex and costly. Micropores clogged by drug residues or tissue fragments are also a potential risk that may disrupt the drug delivery process.

#### 2.1.5 Hydrogel MNs

Hydrogel MNs are fabricated from hydrogel polymer matrices containing three-dimensional (3D) cross-linked networks capable of absorbing water. Upon insertion into target tissues, the MNs swell without dissolving, enabling the drug to be released in a sustained manner [28]. This characteristic not only ensures prolonged drug delivery, but also adds a layer of safety. When an adverse reaction occurs, the hydrogel MNs can be removed immediately. This operation can stop the drug delivery thus avoiding the risks that come with uncontrolled release. These MNs are typically produced by photopolymerization and 3D printing. In photopolymerization, UV light is used to initiate the cross-linking of liquid prepolymers [29], whereas 3D printing enables the layer-by-layer deposition of hydrogel materials. This procedure often works faster than traditional soluble MN fabrication [30]. Commonly used hydrogel materials include polyacrylamide and poly(N-isopropylacrylamide), primarily owing to their biocompatibility and reliable release kinetics. Moreover, researchers have developed bioresponsive hydrogel MNs to achieve intelligent, on-demand drug release in response to physiological cues [31]. In the management of chronic ocular conditions, diffusion-controlled drug release from hydrogel networks enables continuous treatment delivery over several weeks after one time administration. The reduced dosing frequency significantly enhances patient compliance.

#### 2.1.6 Cryo-MNs

Cryo-MNs are a novel MN system fabricated using cryogenic molding techniques, in which the drug is combined with a cryoprotectant, and ice crystals serve as structural support. Owing to their unique physical properties, cryo-MNs remain mechanically rigid at low temperatures, whereas upon insertion into target tissues, the ice crystals gradually melt as the local temperature rises, enabling controlled and precise drug release [32]. The primary method for fabricating cryo-MNs is rapid freezing, whereby the formulation is injected into MN molds and rapidly frozen under low-temperature conditions to form a uniform ice crystal structure that constitutes the MN body [33]. Commonly employed matrix materials include glycerol and PEG, which exhibit favorable molding characteristics at low temperatures and rapidly dissolve at physiological temperatures. Cryo-MNs are particularly suited for delivering substances that are challenging to administer via other MN platforms, such as live cells and large biomacromolecules requiring cryogenic preservation [34]. However, their manufacturing and storage necessitate strict cryogenic conditions to prevent premature melting and preserve drug stability. Although studies investigating the application of cryo-MNs in ocular therapies are currently lacking, their cryogenic properties offer the potential to induce localized anesthesia by temporarily numbing ocular nerves during insertion, suggesting promising applications in ocular anesthesia and pain management. In the future, cryo-MNs may provide a novel technological approach to enhance patient comfort during ocular drug administration while enabling precise and controlled drug delivery.

### 2.2 MNs to overcome the physiological barrier of the eye

**Fig. 5:**
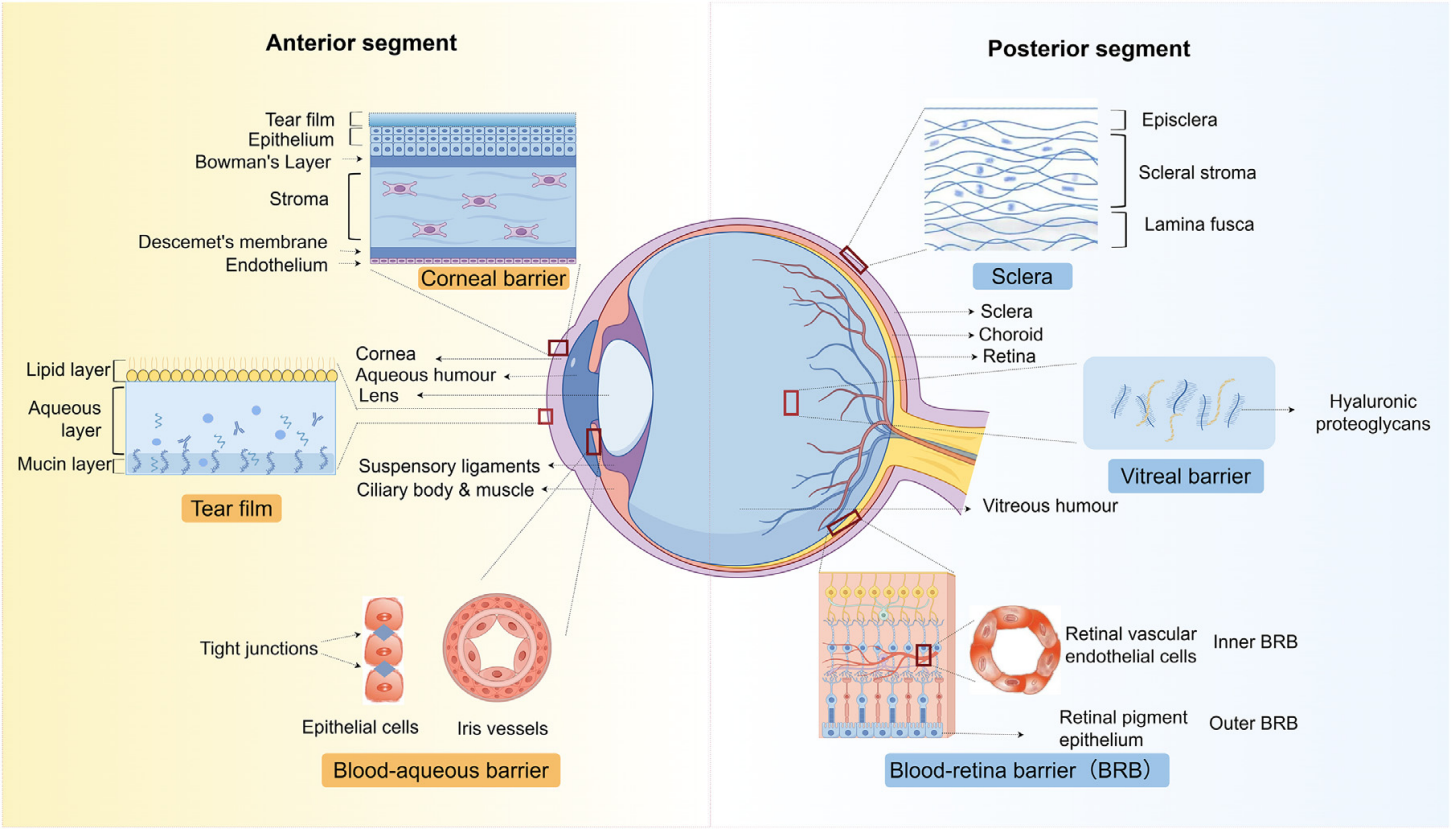
Ocular anatomy and physiological barriers. (By Figdraw).

Physiological barriers, including the tear film and the corneal, blood-aqueous, scleral, vitreous and BRB, impede the delivery of drugs from the external environment to intraocular tissues, as shown in Fig. 5. While these barriers play a crucial protective role by shielding ocular tissues from external threats, they also make delivering drugs to the inside of the eye remarkably difficult. For instance, the tear film quickly flushes away eye drops, shortening their contact time with the ocular surface. Corneal epithelial cells are tightly packed, resulting in severely limited drug penetration by this epithelial cell layer. The blood-aqueous and BRB selectively restrict the transport of drugs from systemic circulation into intraocular tissues. Sclera contains dense and fibrous structures. They create a physical hurdle to limit drug permeation. Vitreous humor is highly viscous and has low fluidity. Its gel-like consistency and slow turnover further impede drug movement in the back of the eye. A comprehensive understanding of these ocular barriers and their influence on drug pharmacokinetics is fundamental for researchers to design smarter delivery systems that can overcome these challenges.

#### 2.2.1 Tear film barrier

The tear film is the first major barrier encountered in ocular drug delivery. Produced continuously by the lacrimal glands, this thin fluid layer coats the entire ocular surface. It is composed of water, electrolytes, proteins and lipids [35]. The tear film is organized in three layers: a superficial lipid layer, an intermediate aqueous layer, and an inner mucin layer [36]. The overall thickness of the tear film typically ranges from 3 to 10 µm [37], with functions that ensure lubrication, hydration and protection of the ocular surface. The tear film poses a significant barrier to drug penetration primarily due to its dynamic renewal and rapid clearance properties. It renews itself at a rate of ∼16% per minute [38], rapidly clearing away topically applied drugs, reducing their retention time on the ocular surface. In addition, enzymes such as lysozyme and other proteins present in the tear fluid can break down drug molecules, compromising their stability and bioavailability [39]. These properties make the tear film particularly challenging for the delivery of hydrophobic compounds and large molecular therapeutics, directly impacting the efficacy of treatments for anterior segment diseases like DED, keratitis and conjunctivitis.

To address the challenges of the tear film barrier, researchers have developed a range of drug delivery systems, including nanoparticles, microemulsions, *in situ* gels and drug-eluting contact lenses [40]. While these methods have improved delivery efficiency to some extent, they still have several limitations, such as complex fabrication processes, limited stability, risk of drug leakage, and the potential to cause patient discomfort. In this context, MN technology presents itself as an alternative. MNs are equipped with micro-scale needle tips and are capable of generating microchannels to bypass the tear film entirely, delivering drugs directly to the underlying tissues. This process avoids rapid tear clearance and enzymatic degradation, thereby improving the bioavailability of the drugs. For example, in the management of DED, MNs can effectively deliver artificial tears or anti-inflammatory agents, thereby helping restore the ocular surface and relieve symptoms [41]. In keratitis treatment, antibiotics or antifungals can be administered directly to the infected area, improving efficacy even at lower doses [42]. In case of conjunctivitis, MNs can achieve sustained drug release, thus decreasing the dosing frequency [43].

#### 2.2.2 Corneal barrier

Corneal barrier is among the most critical physiological obstacles in ocular drug delivery and comprises five distinct layers: epithelium, anterior elastic (Bowman’s) layer, stroma, posterior elastic (Descemet’s) layer and endothelium, with an overall thickness of approximately 500–600 µm [44,45]. The outermost epithelial layer consists of tightly connected epithelial cells with hydrophobic properties and tight junctions. It serves as the principal barrier that severely restricts the passive diffusion of most drugs, particularly macromolecules and hydrophobic agents. In addition to the epithelium, the stroma, which is rich in hydrophilic collagen fibers, further impedes the permeation of lipophilic compounds. The innermost monolayered endothelium limits drug penetration into the anterior chamber [46]. This multilayered architecture and diverse physicochemical properties of the cornea collectively pose significant challenges for achieving effective transcorneal drug delivery via passive diffusion, thereby contributing to low ocular bioavailability. Moreover, the inherently low metabolic activity and limited regenerative capacity of the cornea further complicate the development of efficient and safe drug delivery strategies.

The corneal barrier markedly limits the therapeutic efficacy of clinically used drugs. For instance, in the treatment of bacterial keratitis, standard formulations like tobramycin or levofloxacin eye drops are difficult to penetrate deeply into the cornea, often requiring frequent application just to maintain therapeutic concentrations [47]. Similarly, anti-inflammatory agents such as dexamethasone and fluorometholone are often delivered as ophthalmic ointments or suspensions to alleviate corneal inflammation. However, their limited corneal permeability requires prolonged use, which may lead to adverse effects, including elevated IOP or cataract formation. In contrast, MN technology offers a promising alternative by allowing drugs to directly penetrate the corneal surface through the microchannels created by the needles. For example, MNs have been shown to enable the precise delivery of antibiotics or antifungal agents to the infection site in keratitis, significantly enhancing therapeutic efficacy while reducing drug dosage requirements [48]. For conditions like CNV, MNs can provide sustained release of anti-angiogenic agents, effectively inhibiting pathological vessel proliferation and improving visual outcomes [49].

#### 2.2.3 Blood-aqueous barrier

Blood-aqueous barrier (BAB) is a key physiological barrier in ocular drug delivery and is primarily composed of nonpigmented epithelial cells of the ciliary body and retinal vascular endothelial cells [50]. These cells, which are interconnected by tight junctions, exhibit selective permeability that tightly regulates the exchange of substances between the bloodstream and the aqueous humor, thereby maintaining intraocular homeostasis. However, this protective function also severely restricts the penetration of systemically administered drugs into the aqueous humor, particularly for hydrophilic compounds and macromolecules. These therapeutics exhibit limited transcellular diffusion because of their polar characteristics and inability to traverse the lipid bilayer or cellular interstitial spaces [51]. Furthermore, active efflux mechanisms, such as P-glycoprotein (P-gp), reduce drug concentrations within the aqueous humor, substantially compromising ocular bioavailability [52]. This dual-barrier system, coupled with its dynamic regulatory function, makes the delivery of therapeutics to intraocular tissues via systemic routes, such as oral or intravenous administration, highly inefficient.

Therefore, BAB is a critical determinant of ocular drug distribution and therapeutic efficacy. For example, pharmacokinetic studies have demonstrated that baicalin exhibits poor penetration across the BAB following oral administration, with significantly lower concentrations and area under the curve (AUC) in the aqueous humor than in plasma, highlighting the formidable barrier function of the BAB and its impact on therapeutic outcomes [53]. In the management of uveitis, systemic or local administration of anti-inflammatory agents such as prednisolone acetate is frequently employed; however, the presence of the BAB hampers precise control over intraocular drug distribution and concentration, resulting in suboptimal therapeutic efficacy and an increased risk of systemic side effects. MNs technology offers a promising strategy to overcome the limitations imposed by the BAB. By mechanically creating microchannels at localized ocular sites, MNs can transiently disrupt the integrity of the barrier, providing a direct pathway for drug delivery into intraocular tissues. This approach significantly enhances drug permeability, promotes a more uniform intraocular drug distribution, and minimizes systemic exposure, representing a novel and efficient strategy for the treatment of intraocular diseases.

#### 2.2.4 Scleral barrier

Scleral barrier is a critical anatomical and physiological obstacle for ocular drug delivery and is composed predominantly of densely packed collagen and elastic fibers that confer high mechanical strength and low permeability [54]. The scleral thickness varies between 0.3 and 1.0 mm, providing essential mechanical support and protection to the eye; however, this dense fibrous architecture significantly impedes drug permeation, particularly for hydrophilic compounds and macromolecular therapeutics [55]. The low metabolic activity and sparse vascularization of the sclera further hinder drug diffusion and local accumulation, posing substantial challenges for achieving effective drug concentrations in the posterior segment of the eye [56].

To overcome the scleral barrier and enhance intraocular drug delivery, several strategies have been explored. Iontophoresis promotes the transscleral transport of charged drug molecules via the application of an electric field. However, its efficacy is limited by the physicochemical properties of the drugs and the high electrical resistance of the sclera [57]. Similarly, low-frequency unfocused pulsed ultrasound can temporarily increase scleral permeability, but its prolonged use carries the risk of causing tissue damage [58]. Nanocarrier-based delivery systems, including polymeric micelles and liposomes, have also been employed to facilitate transscleral drug delivery by improving drug solubility, enhancing permeability, and extending ocular residence time. Nevertheless, studies have shown that only nanoparticles within the size range of 20–80 nm can effectively diffuse through scleral pores into the vitreous humor, with overall low diffusion efficiencies and potential biocompatibility concerns [59].

In contrast, MN technology offers a promising approach based on physical penetration to overcome the scleral barrier. The micron-scale tips and mechanical robustness of MNs enable efficient penetration through the sclera, conjunctiva and associated ocular tissues, allowing direct drug administration to the vitreous or posterior segment with minimal invasiveness and reduced adverse effects. Except for small molecule drugs, MNs also show great potential for delivering larger therapeutics. For instance, they can be used to administer hydrophilic drugs, proteins and even nucleic acids into the suprachoroidal space (SCS) to treat conditions like non-infectious uveitis. Notably, studies using MNs to deliver adeno-associated virus serotype 8 (AAV8) vectors into the SCS and subretinal spaces have demonstrated successful ocular gene expression. The specific injection site was found to influence the expression pattern and the inflammatory reaction [60]. Together, these findings highlight the role of MNs as effective and versatile carriers to bypass the scleral barrier.

#### 2.2.5 Vitreous barrier

Vitreous barrier presents a major challenge for delivering drugs to the back of the eye. It is made up of two key components: a gel-like vitreous matrix and the vitreoretinal interface. The matrix contains 98%−99% water and a network of collagen fibers and HA, thus a dense and viscous meshwork is created, which severely limits the movement of drugs, especially larger molecules and hydrophilic compounds [61]. In addition, negatively charged HA interacts readily with positively charged drug molecules, slowing their diffusion even further. The vitreoretinal interface consists of the tightly coupled internal limiting membrane (ILM) and retinal pigment epithelial (RPE) cells. This extra structural barrier in the vitreous blocks drugs from efficiently reaching the retina. The vitreous matrix and vitreoretinal interface pose a major obstacle for drugs to reach their therapeutic concentrations in the retina and choroid, whether through local or systemic administration [62]. Moreover, drugs can also be broken down by enzymes within the vitreous. Coupled with slow metabolic turnover and poor clearance capacity of vitreous humor, drug delivery is further restrained [63]. Such barrier properties especially complicate the management of posterior segment diseases such as DR, AMD and RVO. However, conventional modalities, like intravitreal injection (IVT), often fall short in both safety and efficacy.

To address such issues, various long-acting drug formulations and delivery strategies have been developed to enhance therapeutic efficacy and patient compliance. Dexamethasone intravitreal implants (Ozurdex®) can ensure sufficient drug efficacy within 3–6 months [64], whereas fluocinolone acetonide implants (Retisert® and Iluvien®) can maintain therapeutic levels in the body for up to 2–3 years [65]. These formulations are commonly delivered via IVT or intravitreal implantation, both of which produce high local concentrations and sustained efficacy. Despite this, invasive injections are still in common use. Intravitreal implants require surgery to place the device into the vitreous and may eventually need removal. The risk is especially high in retinoblastoma (Rb) treatment, where withdrawing the syringe needle can cause tumor spreading outside the eye [66]. Consequently, developing non-invasive alternatives is in urgent demand to treat those diseases.

Compared to a standard syringe needle, MNs are extremely thin and make temporary microchannels with minimal ocular tissue damage. By loading hydrophilic drugs and macromolecular biologics in MNs, this technology allows those therapeutics to be efficiently and directly delivered to the posterior segment, while avoiding the invasiveness and associated risks of conventional methods. By blending efficacy with a gentle procedure, MN based delivery could significantly improve treatment safety, enhance therapeutic outcomes, and encourage greater adherence among patients requiring long-term therapy for vision-threatening RD.

#### 2.2.6 Blood-retinal barrier

Blood‒retinal barrier (BRB) is among the most formidable physiological barriers in ocular drug delivery. It plays a critical role in maintaining the stability of the intraocular microenvironment. BRB is a dual layer system. The inner BRB is formed by retinal vascular endothelial cells interconnected by tight junctions. The function of inner BRB is to regulate the exchange of substances between the bloodstream and retinal tissue. The outer BRB is made up of RPE cells. This second layer of BRB further restricts the diffusion of compounds from the choroidal circulation into the retina via tight junctions and active transport mechanisms [67,68]. While this dual-barrier system effectively protects the retina from systemic toxins and pathogens, it also poses a significant challenge for the delivery of therapeutics, particularly macromolecules and hydrophilic drugs. Compounds with molecular weights exceeding 400 Da and hydrophilic agents typically achieve penetration rates of less than 5% [69]. This restrictive characteristic of the BRB complicates the pharmacological management of RDs such as retinopathy, AMD and DR, all of which often necessitate long-term treatment.

In recent years, researchers have devoted great efforts to find ways to overcome BRB, and the results so far are encouraging. For instance, Guo et al. [70] successfully delivered small interfering RNA (siRNA) encapsulated within a triblock copolymer via intravitreal injection, achieving BRB penetration and inhibition of neovascularization. Similarly, Zuo et al. [71] employed subconjunctival injection of tetrahedral framework nucleic acids (tFNAs) conjugated with anti-vascular endothelial growth factor (anti-VEGF) aptamers, which effectively penetrated the outer BRB and reached the inner retina, demonstrating potent anti-angiogenic effects in both *ex vivo* and *in vivo* models. Furthermore, Kang et al. [72] designed a smart light-responsive nanocarrier system that is administered systemically. Upon localized light activation, the drug is precisely triggered to be released within the retina. This strategy effectively bypasses the BRB, enabling focused treatment of Rb. However, challenges related to long-term safety, delivery efficiency and practical clinical application still need to be addressed.

Within this context, MN technology represents a versatile and minimally invasive platform. MNs can bypass BRB entirely. MNs can instantly create microchannels through the sclera to the SCS or the peripheral vitreous cavity, providing its payloads immediate access to the RPE. Once the drug is deposited, a strong concentration gradient rapidly forms. The therapeutic agent passively diffuses, taking either transcellular or paracellular routes, across the RPE and continues moving into the neurosensory retina. This enables precise targeting of key cells, including photoreceptors, bipolar cells, and retinal ganglion cells. In the meantime, the micro-incision induced by MN insertion self-seals within minutes, preventing prolonged leakage or significant bleeding. Moreover, this technology is highly tunable. MN materials, needle geometry, and drug-release kinetics can be customized according to individual diseases or conditions. This opens the door to patient-specific treatment regimens tailored to various retinal-disease subtypes. By offering a direct, localized, and adjustable drug delivery, MNs represent a promising strategy to bypass the BRB.

### 2.3 Design of MNs

The design of MNs for ocular drug delivery requires careful consideration on material selection, geometric configuration, substrate design and dimensional parameters. All of these factors directly impact the mechanical performance, drug-release efficiency, ocular tissue compatibility and overall clinical safety of the MN system.

#### 2.3.1 Materials and mechanical properties

Material selection is one of the most important steps in designing MNs for ocular drug delivery. The property of materials directly influences the mechanical strength, drug-releasing capability, and clinical safety of MNs. To meet the specific requirements of the eye, several key characteristics, such as mechanical robustness, drug-release kinetics, biocompatibility and ocular-tissue compatibility, must be carefully evaluated before clinical use. In the case of corneal implantation, optical transparency is also required. Currently, materials used in MN fabrication can be grouped into 3 categories: inorganic materials, natural biomaterials and synthetic polymers.

Inorganic materials used for MN fabrication include nondegradable materials such as metals, silicon and glass. Among these, metal-based MNs are widely utilized in biomedical applications because of their well-established fabrication techniques, excellent ductility, superior mechanical strength, and ability to efficiently penetrate target tissues [73]. In contrast, glass MNs are typically produced via manual stretching methods, which are associated with several drawbacks, including low production efficiency, poor batch consistency, and the risk of fracture during tissue insertion, potentially eliciting foreign body reactions and thereby limiting their clinical applicability [74]. Natural biomaterials commonly employed for MN fabrication include polysaccharides and proteins. Frequently used polysaccharides, such as HA, chitosan (CS) and PVA, offer excellent biocompatibility and moldability. However, their high hygroscopicity can compromise the structural stability of MNs and may adversely affect drug activity, necessitating strict environmental control during storage and application [75]. Recent studies have increasingly focused on plant-derived polysaccharides, such as astragalus polysaccharide [76] and hyacinth polysaccharide [77]. Both of them possess structural support and biological activity. Upon MN penetration into the cornea or sclera, these natural polysaccharides rapidly absorb tissue fluid and swell, forming a viscoelastic hydrogel that seals the microchannels while exerting antioxidant [78] and wound-healing effects [79]. Their anti-inflammatory properties further downregulate the expression of inflammatory mediators such as IL-6 and TNF-α, thereby mitigating potential inflammation induced by MNs [80]. Subsequently, the dissolved polysaccharides can form an “*in situ* drug reservoir” within the eye. For instance, CS MNs can provide a therapeutic effect for up to 8 d [81], significantly prolonging drug retention and enhancing bioavailability. Additionally, the anionic/cationic groups carried by polysaccharides can engage in electrostatic interactions with corneal epithelial mucins, further extending drug residence time in the eye. These collective characteristics endow polysaccharides with unique advantages as MN materials for ocular drug delivery. Protein-based materials, including sericin, gelatin and collagen, are also popular for ocular drug delivery applications because of their favorable processability and biocompatibility [82]. On the synthetic front, polymers such as polylactic acid (PLA), polylactic-co-glycolic acid (PLGA), and polyvinylpyrrolidone (PVP) have emerged as materials of choice for MN fabrication. The mechanical strength can be finely tuned, and those materials can break down in the body at a predictable rate [83]. These advantages contribute to precise control over drug release, whether on the eye’s surface or deep within the intraocular environment. As these polymers are biodegradable, the needles would safely dissolve away over time, avoiding surgical removal, which may lead to infection.

In addition to the general requirements for MN applications, designing MNs for the eye comes with its unique set of challenges. The materials must have sufficient mechanical strength to prevent any breakage when penetrating the ocular tissues. For corneal MNs, transparency is essential to avoid obstructing the patient’s vision after application. Therefore, the selection of materials for ocular MNs must consider parameters such as biocompatibility, mechanical strength, degradation kinetics, optical properties and other functional requirements. The goal is to optimize these material properties to create a system that delivers drugs not just efficiently, but also safely and with precision.

#### 2.3.2 Geometric design

The sizes and shapes of ocular MNs play an important role in determining drug delivery effectiveness, thereby necessitating careful consideration of ocular anatomy, delivery efficiency, and clinical safety. Generally, MN is made up of two key parts: a tip and a base. The synergistic effect between the tips and the base mainly influences the penetration ability, drug loading capacity, and usage effect of MN. Conical or prismatic shapes are commonly used for MN tips because those geometries require low insertion force. Sharp tips are embedded into ocular tissues more easily, but concentrate mechanical stress in a very small area, bringing greater risks of causing tissue injuries. A moderately blunt tip is used to balance the penetration effect with the tissue damage risk. To optimize both delivery efficiency and drug loading capacity, composite tip structures integrating a tapered tip with a cylindrical body have been developed. This hybrid configuration preserves penetration efficacy while substantially enhancing drug loading. For hollow MNs, the tip geometry plays a pivotal role in minimizing insertion resistance and regulating drug flow rates. Adjustments to the taper angle and outer diameter can optimize both penetration dynamics and infusion efficiency. Notably, a larger tip bevel angle may facilitate improved tissue penetration and drug dispersion; however, it also carries an elevated risk of leakage, rendering it more suitable for specific applications such as SCS injections. In recent years, biomimetic design strategies have offered innovative approaches to MN development. For example, MNs inspired by the vibrating, serrated structural features of mosquito mouthparts have been shown to significantly reduce tissue penetration resistance [84,85]. Similarly, designs mimicking the adhesion mechanisms of the blue-ringed octopus sucker [86], the dentition of leeches [87], and the stinging apparatus of honeybees [88] have been explored to enhance MN adaptability to the unique physiological characteristics of ocular tissues, thereby enabling more precise, efficient, and less traumatic drug delivery.

**Fig. 6:**
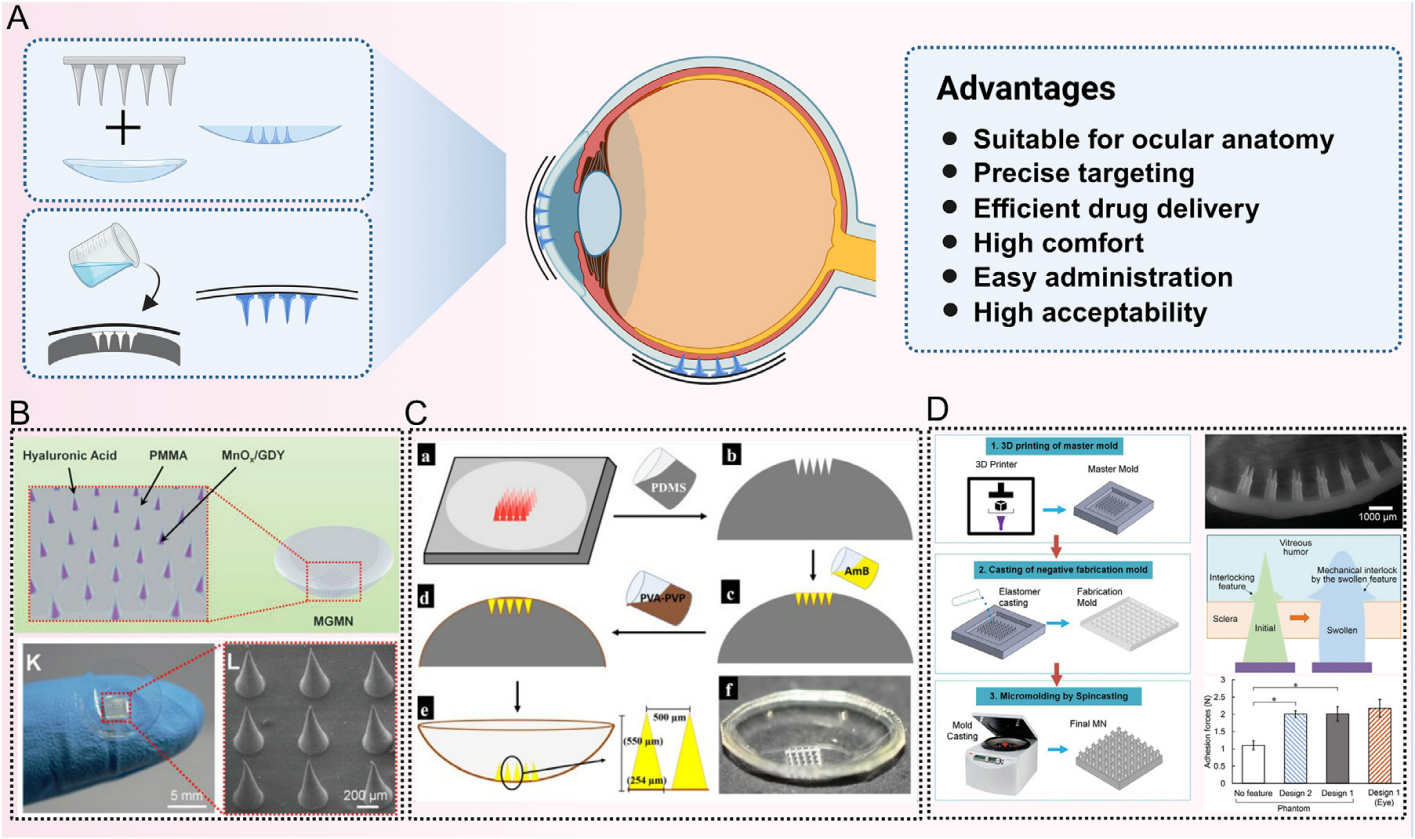
Fabrication and application of curved MNPes for ocular drug delivery. (A) Schematic illustration of the fabrication and application process of curved-base MNs; (B) Characterization of MnOx/GDY and MGMN. Reprint with permission from [114]. Copyright© 2023 Wiley-VCH GmbH; (C) Fabrication process of curved-base PVA-PVP MNs. Reprint with permission from [116]. Copyright© 2019 Elsevier B.V.; (D) Fabrication of curved base MNs and their characterization. Reprint with permission from [89]. Copyright© 2020 WILEY-VCH Verlag GmbH & Co. KGaA, Weinheim. (Created with BioRender.com).

The design of the MN base is equally crucial in determining the overall therapeutic outcome, as it influences factors such as drug distribution, release efficiency, tissue compatibility and patient comfort (Fig. 6D) [89]. A poorly designed base can result in uneven drug distribution, slower release rates, irritation to ocular tissues, and even visual disturbances. Therefore, optimizing the MN base structure to conform to ocular anatomical requirements and intended use scenarios can improve the efficacy and safety of MN-mediated ocular drug delivery.

In view of the geometric configuration, it is essential to take into account the biomechanical characteristics of the MN substrate to ensure optimal adaptation to the curved ocular surface, minimal tissue trauma, and improved drug-delivery efficacy. Fig. 6 presents the fabrication and application of some curved-base MN patches (MNP) for ocular drug delivery. Different design strategies are required for various ocular target regions. For instance, flat substrates are appropriate for drug delivery to the relatively planar conjunctival area, whereas curved substrates are better suited for applications on the cornea and sclera, where they can improve interfacial conformity and reduce mechanical mismatch [90]. This bio-inspired curvature design not only minimizes tissue damage caused by friction between the MN array and the ocular surface, but also ensures more uniform needle insertion across the array, thereby significantly enhancing drug delivery efficiency. Additionally, rounding the edges of the MN base can reduce irritation to sensitive ocular tissues, while incorporating flexibility and elasticity into the base material can alleviate local compression, reduce the risk of tissue injury, and improve wearing comfort.

In terms of array configuration, round or oval-shaped MNPs are preferred for localized, lesion-specific drug delivery, whereas stripe- or grid-patterned arrays are more conducive to achieving uniform drug penetration over larger therapeutic areas. Furthermore, optimal tip density is a critical design parameter in ocular MNPs. Although higher tip densities can increase drug loading capacity, they are associated with increased risks of ocular tissue injury, inflammation and local drug accumulation, which may accelerate premature drug clearance due to tear washout. High-density arrays also increase foreign body sensation, insertion resistance and manufacturing complexity, potentially resulting in difficulties during application or removal, tip adhesion and mechanical failure. Conversely, excessively low tip densities may compromise drug delivery efficacy, fail to achieve therapeutic concentrations, and weaken the structural stability of the MN array, leading to displacement or detachment. Studies have demonstrated that when the tip density falls below 5 needles/mm², the adhesion duration is reduced by more than 30% [91]. Therefore, the design of ocular MN tip density must carefully balance drug delivery efficiency, mechanical stability, and ocular tissue tolerance to optimize therapeutic outcomes.

#### 2.3.3 Dimensional design

The dimensional design of ocular MNs must be precisely tailored to the anatomical and physiological characteristics of the target ocular tissues, with particular attention to tip length, base dimensions and adjustments based on the specific animal models employed in preclinical studies [92]. Table 2 summarizes key ocular parameters of human and experimental animals. For example, the central thickness of the human cornea is approximately 500–550 µm [93], with the epithelial layer measuring 50–60 µm [94]. Accordingly, MN tip lengths of 200–400 µm are generally considered appropriate to ensure sufficient penetration through the corneal epithelium and facilitate effective drug delivery while avoiding inadvertent injury to deeper corneal structures. In contrast, the sclera is thicker, ranging from 300 to 1000 µm [95], and its inherent elasticity further limits complete MN insertion. Therefore, scleral MNs typically require tip lengths of at least 500 µm to ensure adequate drug deposition in deeper ocular compartments, such as the retina and vitreous body. In addition, the diameter of ocular MNs is markedly smaller than that of conventional IVT needles (typically 25–30 gauge, corresponding to 250–300 µm), with MN diameters typically ranging from 50 to 200 µm [96]. This miniaturized design enables targeted drug delivery for diverse therapeutic applications, including corneal repair and retinal anti-inflammatory therapies, while minimizing mechanical trauma to ocular tissues. Moreover, the design of the MN base must also consider the delicate vascular architecture of the eye. Given the rich vascularization and fine distribution of ocular blood vessels, MN substrates should be designed to minimize disruption to peripheral vasculature, thereby reducing the risk of hemorrhage and ensuring safe and effective clinical application.

**Table 2:** Ocular parameters of human and experimental animals.

Species	Eye diameter	Central corneal thickness (CCT)	Scleral thickness
Human	24 mm [177]	0.5–0.55 mm [93]	0.3–1 mm [95]
Pig	20–24 mm [178]	0.8–1.1 mm [179]	0.4–1.2 mm [180]
Rabbit	16 mm	0.35–0.45 mm	0.2–0.5 mm
Rat	6–7 mm [181]	0.15–0.2 mm	0.1–0.3 mm
Mouse	3–4 mm [182]	0.1–0.15 mm	0.05-0.2 mm

## 3 Application of MNs in the treatment of anterior segment eye diseases

Anterior segment diseases, including conjunctivitis, dry eye, corneal diseases, glaucoma and cataract, typically require topical drug administration for effective management. However, conventional ocular drug delivery methods are often associated with low bioavailability, limited tissue penetration and poor patient compliance. Therefore, these methods fail to fully meet clinical therapeutic needs. In recent years, owing to its unique transdermal and transmucosal delivery mechanisms, MN technology has demonstrated considerable potential in addressing these limitations, particularly for the management of acute and immediate-phase ocular diseases. Table 3 summarizes the recent advances of using MNs to treat anterior segment diseases. By enabling precise and localized drug delivery directly to lesion sites, MNs can significantly increase drug bioavailability while minimizing systemic exposure and associated side effects. Moreover, the minimally invasive nature of MNs reduces tissue trauma and discomfort, rendering them especially suitable for application in sensitive ocular regions. Additionally, MN technology can be integrated with adjunctive treatments such as laser therapy and surgical interventions, offering personalized and comprehensive therapeutic strategies tailored to various clinical scenarios.

**Table 3:** Summary of recent advances in ocular anterior segment diseases treatment by MNs.

Disease model	Modeling method	MN type and characteristics (Tip shape, Array, Length)	Animal model/Application site	Functions	Effectiveness	Ref.
Conjunctivitis	Ovalbumin (OVA) and aluminum hydroxide (AH) induced	Soluble MNs Quadrangular, square, 25 needles, 500 µm	New Zealand male white rabbit/ Conjunctiva or cornea	Efficient penetration, precise targeting, minimally invasive, painless	Reduced levels of inflammatory markers such as IgE, TNF-α and IL-6 and increased expression of anti-inflammatory factors such as TGF-β and IL-10, reducing inflammation and tissue damage.	[43]
DED	High-fat diet-induced	Soluble MNs Conical, round, 385 needles, 500 µm	Male BALB/c mice/ Mouse eyelid skin	Efficient penetration, precise targeting, minimally invasive, painless	Increased ROSI levels in mouse leptomeningeal glands, improved tear secretion, reduced corneal epithelial defects, and attenuated leptomeningeal inflammation and tissue damage.	[109]
Bacterial keratitis	Injection of *S. aureus* into the corneal stroma	Core-shell MNs Conical, square, 4 × 4, 850 µm	SD male rats/ Rat corneal stroma	Efficient penetration, precise targeting, minimally invasive, painless	Significantly reduced the number of inflammatory macrophages and neutrophils in the cornea and retina	[113]
	Intra-stromal injection of *P. aeruginosa*	Hydrogel MNs Conical	New Zealand white rabbit/ Corneal surface	Efficient penetration, precise targeting, minimally invasive, painless	Reduced bacterial survival, modulation of macrophage M1/M2 phenotype switching, inhibition of inflammatory response, promotion of corneal repair	[115]
Fungal keratitis	Injecting *C. albicans* suspension into the corneal stroma	Soluble MNs Tapered, contact lens type, MN arrays arranged in the center	New Zealand white rabbit/ Corneal surface	Efficient penetration, precise targeting, minimally invasive, painless	*In vitro* inhibition rate of *Candida albicans* was 99.99%	[114]
	Intracorneal injection of *Candida albicans*	Soluble MNs Pyramidal, contact lens type, 5 × 5 MNs arrays arranged in the center, 550 µm	New Zealand white rabbit/ Corneal surface	Efficient penetration, precise targeting, minimally invasive, painless	Significantly reduced the number of *Candida albicans* in the cornea, resulting in a significant reduction in colony-forming units and improved corneal tissue repair.	[116]
	Intracorneal injection of *Candida albicans*	Soluble MNs Tapered, square, 10 × 10, 700 µm	New Zealand white rabbit/ Corneal surface	Efficient penetration, precise targeting, minimally invasive	Significantly reduced fungal colonization in the cornea	[118]
	Injection of *Fusarium solani* solution into the corneal stroma	Soluble MNs Pyramidal, square, 20 × 20, 390 µm	New Zealand white rabbit/ Corneal surface	Efficient penetration, precise targeting, minimally invasive, painless	Increased drug concentrations in the aqueous humor and cornea, with significant reductions in corneal tissue damage and inflammatory cell infiltration	[176]
	Injection of *Candida albicans* suspension into the corneal stroma	Bilayer soluble MNs Conical, square, 11 × 11, 600 µm	Enucleated pig eye/ Corneal surface	Efficient penetration, precise targeting, minimally invasive, painless	Demonstrated superior antifungal activity in both *in vitro* and *ex vivo* assays, significantly reducing corneal fungal colony-forming units.	[119]
Echinococcus amebic keratitis	Injection of *Acanthamoeba Castellanii* suspension into the corneal stroma	Separable Hybrid MNs Octagonal, cone, single, 150 µm	Female C57BL/6 J mice/ Within the corneal stroma	Efficient penetration, precise targeting, minimally invasive, painless	The number of amebic pathogens in the cornea was significantly lower than in the control group, and corneal transparency was significantly improved after treatment.	[183]
CNV	Defect on the cornea, then dropped S. aureus for infection	Dual-layer MNs Cone, 4 × 4, 850 µm	SD rat/ Corneal surface	Efficient penetration, precise targeting, minimally invasive, painless	Expression levels of inflammatory markers ( TNF-α and IL-6) in the cornea were significantly reduced, and the area of corneal neovascularization was reduced by ∼ 90%.	[125]
	No. 7–0 silk suture was placed ∼1 mm from the corneal limbus, and the silk thread was left in the cornea to continuously stimulate the cornea and induce neovascularization from the limbus to the center	Coated MNs Taper, single, 400 µm	New Zealand white rabbit/ Corneal stroma	Efficient penetration, precise targeting, minimally invasive, painless	Neovascularization at post-treatment Day 18 was reduced by 44%, significantly better than the untreated group	[126]
	10–0 nylon suture was placed ∼1 mm from the corneal limbus, and the thread was left in the cornea to continuously stimulate the cornea and induce neovascularization from the limbus to the center	Solid MNs Pyramidal, single, 140 µm	C57BL/6 J mice/ Corneal stroma	Efficient penetration, precise targeting, minimally invasive, painless	Significant inhibition of corneal neovascularization and reduction in the length and area of neovascularization	[19]
	Alkali burn induction	Dual-layer MNs Pyramidal, 3 × 3, 500 µm	C57BL/6 J mice/ Corneal	Efficient penetration, precise targeting, minimally invasive, painless	Reduced the area of neovascularization by ∼ 90%, significantly reduced the infiltration of F4/80-positive macrophages in the cornea and reduced the levels of IL-6 and VEGF in the tear fluid.	[127]
Glaucoma	Untreated	Hollow MNs Beveled hollow point, single, 700–800 µm	New Zealand white rabbit/ Superciliary cavity	Efficient penetration, precise targeting, minimally invasive, painless	A decrease in intraocular pressure for at least 6 h.	[27]
	Untreated	Hollow MNs Tip is ground to a 60° beveled surface, single, 750 ± 50 µm	New Zealand white rabbit/ Superciliary cavity	Efficient penetration, precise targeting, minimally invasive, painless	Achieve IOP lowering for up to 1 month with a high degree of safety and compliance	[131]
	Untreated	Hollow MNs Tip is ground to a 60° beveled surface, single, 650–750 µm	New Zealand white rabbit/ Suprascleral cavity	Efficient penetration, precise targeting, minimally invasive, painless	Prolongs the effect of lowering IOP for up to 4 months	[132]
	Untreated	Hollow MNs Tip angled at 45, single, 800 µm	Freshly removed rabbit eye/ Hypervascular space	Efficient penetration, precise targeting, minimally invasive, painless	Causes varying degrees of SCS dilation, potentially resulting in varying degrees of IOP reduction	[133]
Conical cornea	After epithelium removal, cotton pads containing 5% type II collagenase were applied for 20 min to induce corneal dilation.	Multi-layer responsive porous MNs Conic, 11 × 11, 400 µm	New Zealand white rabbit/ Corneal surface	Efficient penetration, precise targeting, minimally invasive, painless	Maximum corneal refraction in the microneedle group was reduced by ∼2.1 D after crosslinking. Corneal edema returned to preoperative levels within 1 week after surgery.	[138]
	Type II collagenase induced corneal dilation	Soluble MNs Pyramidal, different numbers of tips for different shapes, 800 µm	New Zealand white rabbit/ Corneal stroma	Efficient penetration, precise targeting, minimally invasive, painless	Significantly reduce the maximum refractive error of the cornea, improve the irregularity of the cornea, and promote the recovery of vision.	[23]

### 3.1 Conjunctivitis

Conjunctivitis is an inflammation of the conjunctiva and is commonly characterized by ocular redness, itching, excessive discharge, foreign body sensation, and tearing [97]. Based on etiology, conjunctivitis can be classified as infectious (bacterial or viral) or non-infectious (allergic or chemical irritation) [98]. Current treatments typically involve topical medications such as eye drops or ointments [99]. However, prolonged use of certain agents, including corticosteroids, may result in adverse effects such as elevated IOP and cataract formation, while the overuse of antibiotics increases the risk of antimicrobial resistance, complicating treatment strategies.

In the management of viral conjunctivitis, agents such as ribavirin, ganciclovir and interferons are commonly employed but are associated with significant side effects. Notably, Zika virus (ZIKV) infection can cause neuro-ocular manifestations, including conjunctivitis, through direct viral invasion of ocular tissues, resulting in conjunctival vasodilation and inflammatory cell infiltration [100]. Jacob et al. [101] demonstrated that MNP vaccination offers significant advantages over conventional intramuscular injection, not only by reducing ocular viral loads and enhancing the production of high-titer, high-affinity neutralizing antibodies but also by attenuating autoimmune ocular pathology through modulation of autoimmune responses. These findings suggest that MN-based vaccination may offer a dual benefit in both preventing viral conjunctivitis and mitigating ZIKV-induced ocular damage, providing a promising strategy for the prevention and management of viral conjunctivitis.

Allergic conjunctivitis (AC) is a hypersensitivity reaction triggered by allergens such as pollen, dust mites and animal dander, with symptoms including itching, redness, tearing and, in severe cases, conjunctival edema and vision impairment [102]. Elhabal et al. [43] developed a novel therapeutic regimen (KF-SP-MNs) combining a nanodelivery system in MN to overcome poor drug penetration and low bioavailability in AC treatment. PVP/PVA was selected as the MN tip material and internally loaded with nanocarrier-encapsulated ketotifen fumarate (KF), a drug with dual antihistamine and mast cell stabilization [103]. KF was first encapsulated in nanocarriers to enhance its solubility and stability, and then embedded into the MN matrix. This design allows the MNP to penetrate the corneal surface, enabling targeted and sustained drug release. The results of *in vitro* and *in vivo* experiments revealed that the MNP achieved 93% cumulative drug release within 72 h, with good mechanical properties. Compared with other treatment groups, KF-SP-MNs showed stronger anti-inflammatory and immunomodulatory effects *in vivo*, significantly reducing the levels of inflammatory markers of IgE, TNF-α and IL-6, but increasing the expression of the anti-inflammatory factors of TGF-β and IL-10, offering pronounced relief from the symptoms of AC. In addition, histopathologic examination confirmed that KF-SP-MNs rapidly restored the normal structure of the conjunctiva and cornea, reducing inflammation and tissue damage.

### 3.2 Dry eye diseases

Based on the underlying etiology, dry eye diseases (DED) can be classified into two types: hyposecretory dry eye, which is caused by decreased tear production, and hyperevaporative dry eye, which often results from meibomian gland dysfunction (MGD) or blepharospasm dysfunction [104]. Current treatment modalities for DED include artificial tears, anti-inflammatory medications such as cyclosporine A (CsA) eye drops, and physical therapies such as hot compresses and blepharoplasty [105]. However, prolonged use of artificial tears may mask the underlying etiology, whereas excessive use of anti-inflammatory medications can lead to ocular irritation or side effects, including elevated IOP or increased infection risk. Therefore, the development of novel, effective, and safe therapeutic strategies for DED is critical.

Unlike traditional eye drops, MN drug delivery systems provide significant advantages by reducing drug loss and enabling sustained release at the target site, thereby improving therapeutic efficacy. Biodegradable MN acupuncture (BMA) has emerged as a promising treatment for DED, offering comparable efficacy to traditional intradermal acupuncture (IDA) with improved safety [106,107]. Research shows that BMA can effectively stimulate key acupoints around the eye, like BL2 and GB14. This promote local blood circulation and neuromodulation, which helps boost natural tear production. Additionally, it eliminates the risk of metal allergic reactions linked to IDA, providing a valuable alternative for patients with metal allergies.

**Fig. 7:**
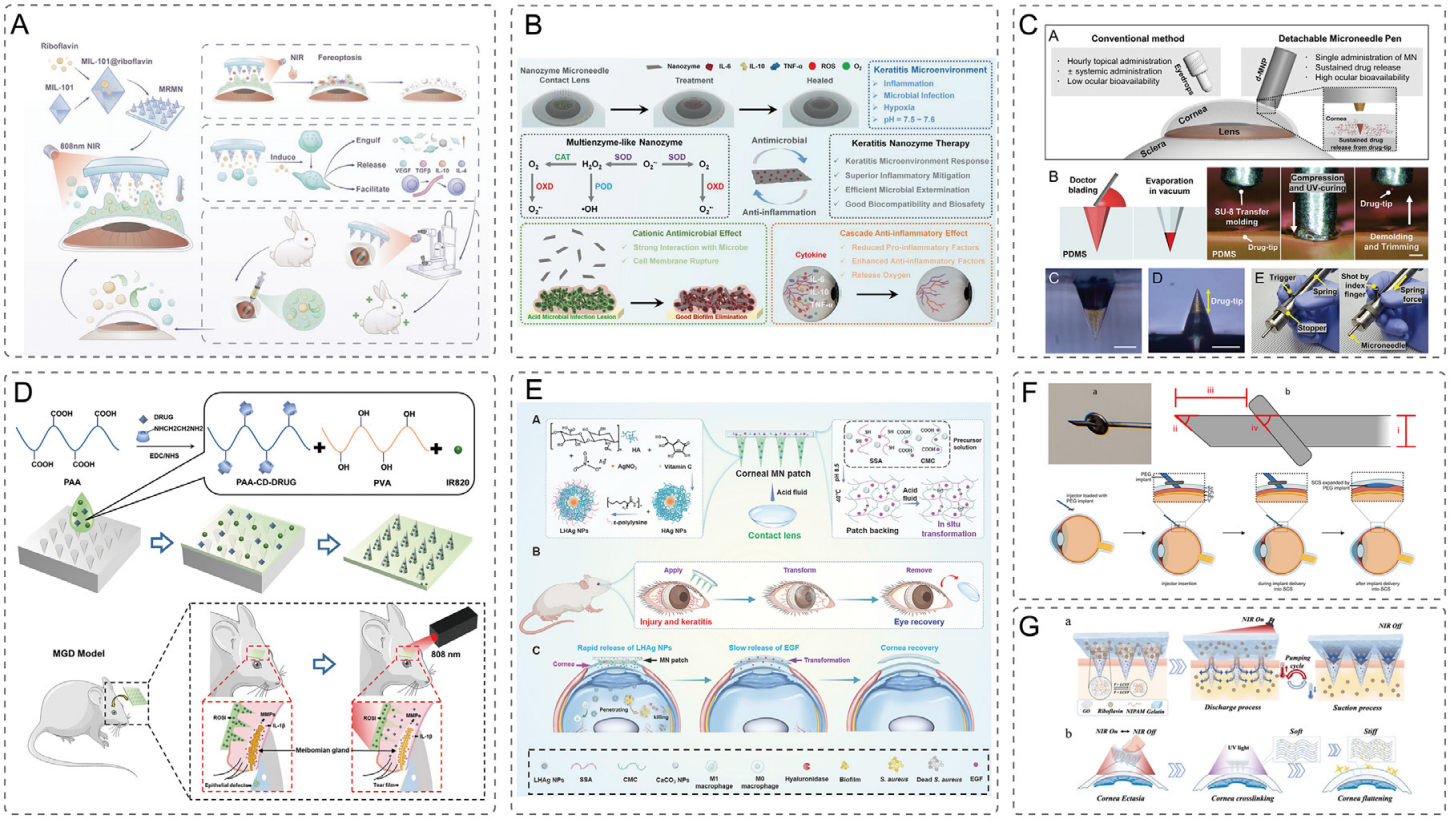
Recent advances of MNs for anterior segment ocular pathologies. (A) MNs patches combined with thermotherapy for bacterial keratitis. Reprint with permission from [115]. Copyright© 2024 Wiley-VCH GmbH; (B) Therapeutic mechanism of multienzyme-like nanozyme-loaded MNs infectious keratitis therapy. Reprint with permission from [114]. Copyright© 2023 Wiley-VCH GmbH; (C) MNs delivering high-concentration drugs for infectious keratitis therapy. Reprint with permission from [42]. Copyright© 2021 Wiley-VCH GmbH; (D) MNPs under 808 nm laser irradiation for the treatment of MGD. Reprint with permission from[109]. Copyright© 2025 The Author(s); (E) Corneal MNPs for the treatment of ocular injury and infection. Reprint with permission from [125]. Copyright© 2025 The Author(s). . (F) Intraocular precision injection of MNs to treat glaucoma Reprint with permission from [133]. (G) Responsive porous MNs for corneal collagen crosslink. Reprint with permission from [138]. Copyright© 2024, The Author(s).

MGD is a leading cause of evaporative dry eye. Conventional drug delivery methods frequently struggle to penetrate the corneal barrier to achieve sufficient therapeutic concentrations within the meibomian glands [108]. Yu et al. [109] developed a novel photothermally responsive soluble MNP for treating MGD, incorporating both an indocyanine green derivative (IR820) and the peroxisome proliferator-activated receptor gamma (PPAR-γ) agonist rosiglitazone (ROSI). Under near-infrared (NIR) light irradiation, the photothermal effect promoted targeted drug delivery into the meibomian glands following patch insertion through the eyelid. This temperature rise effectively liquefied obstructed meibomian gland lipids and cleared ductal blockages (Fig. 7D). *In vitro* tests, the MNP possessed outstanding physical properties such as toughness and penetration ability, sustaining the drug release for as long as 100 h post implantation. Consistent results were observed in the *in vivo* model, which showed that the ROSI content in the meibomian glands increased significantly when applying the MNP. This contributed to improving the tear production, decreasing corneal epithelial defects, lessening inflammation, and retarding the tissue damage in the meibomian glands. All these results validated that this MNP effectively alleviated MGD-associated dry eye symptoms through efficient drug delivery and photothermal-drug synergy, offering a novel and promising strategy for treating MGD.

### 3.3 Keratitis

Keratitis refers to the corneal inflammation [110]. Based on its etiology, keratitis can be divided into infectious keratitis, such as keratitis caused by bacteria, viruses, fungi, or parasites; and non-infectious keratitis, which may arise from traumas, chemical exposures, dry eyes, and immune reactions [111]. The treatment of keratitis is challenging. Dilution of tears leads to low drug bioavailability of eye drops. This often forces frequent dosing, which can cause side effects and poor patient adherence, resulting in increased risk of treatment failure and complications. Therefore, the development of innovative and effective drug delivery systems and customized therapeutic strategies is required to improve treatment outcomes for this condition.

#### 3.3.1 Bacterial keratitis

*Staphylococcus aureus* (*S. aureus*) is a major causative agent of bacterial keratitis, and its infection leads to a decrease in the pH of the local tissue microenvironment, which exacerbates disease progression [112]. To address this issue, Jiang et al. [113] developed a self-implantable, core-shell structured MNP in which the core was loaded with pH-responsive antimicrobial nanoparticles (Ag@ZIF-8) based on water-soluble PVA. These nanoparticles were capable of rapidly releasing antimicrobial metal ions (Zn²⁺ and Ag⁺) in the acidic environment of the infected cornea. Meanwhile, the outer shell was fabricated from biodegradable silk fibroin (SF) for encapsulating the antiangiogenic drug rapamycin (Rapa). This provides sustained anti-inflammatory and anti-angiogenic effects of Rapa for up to 7 d. *In vivo* experiments showed that the number of *S. aureus* colonies in the corneal tissues of the rats was significantly decreased after MN treatment for 5 d. The bacteria exhibited clear membrane rupture and cytoplasmic leakage. This antibacterial effect was especially strong in acidic conditions (pH 5.6), confirming the acid-responsive release of Rapa from MNs. Importantly, the corneal epithelial and stromal layers stayed clear and intact, indicating the MN treatment was safe.

Similarly, Liu et al. [114] developed a multienzyme-like nanoenzyme system (MGMN) incorporated into a HA and polymethyl methacrylate (PMMA)-based MNP (Fig. 6B). By loading manganese oxide (MnOx) and graphene nanoenzymes into MN matrix, this system demonstrated strong *in vitro* antimicrobial activity against methicillin-resistant *S. aureus*, with 99.99% inhibition rate. MGMN also modulated the immune response. It significantly lowered pro-inflammatory markers of TNF-α and IL-6 and elevated the anti-inflammatory cytokine IL-10. Through the dual action of suppressing inflammation and promoting corneal healing, MGMN demonstrated remarkable potential for bacterial keratitis (Fig. 7B).

Zhou et al. [115] developed a smart MN system (MR@MN) that integrates photothermal therapy and immunomodulation to combat *Pseudomonas aeruginosa (P. aeruginosa)* and *S. aureus-*induced keratitis. In MR@MN, iron-based metal-organic frameworks (MIL-101) and riboflavin composite nanoparticles were incorporated into hydrogel-based MNs (Fig. 7A). After activation by NIR irradiation, the MNP generates a controlled photothermal effect. The localized elevated temperature directly disrupts the structural integrity of bacterial membranes. The burst of ROS induced by the system further contributes to the breakdown of biofilm matrices. Meanwhile, the released Fe³⁺ ions trigger severe metabolic dysfunction by driving lipid peroxidation, leading to bacterial ferroptosis, a form of iron-dependent cell death. *In vitro* studies in a simulated infected microenvironment (pH 5.5) demonstrated that the bacterial survival rate in the MR@MN + NIR group decreased to 17.6% ± 3.3%, approximately 8-fold lower than that observed in the control group. *In vivo* experiments revealed significant improvements in corneal opacity after 7 d of treatment, accompanied by reduced inflammatory cell infiltration and a decrease in the key inflammatory cytokine IL-6. Mechanistic studies indicated that riboflavin, released because of the reactive degradation of MIL-101 in an acidic environment, promoted tissue repair while suppressing excessive inflammation by modulating the M1/M2 macrophage phenotype switch. The MR@MN system demonstrated significant synergistic antibacterial and anti-inflammatory effects through the cascade of "photothermal sterilization-iron-mediated death potentiation-immunomodulation and repair", highlighting its potential as a novel therapeutic strategy for bacterial keratitis.

#### 3.3.2 Fungal keratitis

The contact lens-based MGMN system developed by Liu et al. [114] for the synergistic treatment of fungal keratitis caused by drug-resistant *Candida albicans* integrates a flexible PMMA substrate with an HA MN array. The system features a biomimetic curved substrate (with a curvature radius of 8.6 mm), ensuring a precise fit on the corneal surface and minimizing the mechanical damage often associated with conventional MNs. Upon insertion into the cornea, the HA MNs dissolve within 30 s, forming a long-lasting drug reservoir that continuously releases MnOx/gadolinium yttrium (GDY) nanoenzymes at a sustained release rate of 92% over 24 h. The nanoenzymes exhibit multienzyme activity, achieving the therapeutic effects of sterilization, anti-inflammation, and tissue repair through the regulation of ROS levels. Additionally, the nanoenzymes catalyze oxygen generation, increasing the dissolved oxygen concentration by 2.8 times and significantly improving the hypoxic microenvironment of the cornea. *In vitro* studies demonstrated that the MGMN system was 4.8 times more efficient than voriconazole in eradicating drug-resistant *Candida albicans*, reducing the 24-h survival rate of the pathogen to 8.7%. Furthermore, the system effectively reduced ROS levels and decreased malondialdehyde (MDA) concentrations by 76%. This technology addresses the challenge of low bioavailability (<5%) associated with traditional eye drops and ointments, providing an innovative therapeutic option for drug-resistant ocular surface infections.

Roy et al. [116] turned their attention to fungal keratitis, which is often caused by *Candida albicans*. They developed a soluble MNP composed of PVA and PVP polymers and loaded it with the antifungal drug amphotericin B (AmB) (Fig. 6C). This MNP increased AmB retention in the rabbit cornea 4-fold compared to free drug administration. It also limited the systemic distribution of AmB, significantly reducing the associated risk of systemic toxicity. These findings highlight that MN technology is not just applicable for bacterial infections, but also an effective and safe platform for treating fungal eye diseases as well.

Fluconazole (FNL) is a potent antifungal agent that is commonly used against fungal keratitis [117]. However, the effectiveness of FNL is often hampered by its poor water solubility and difficulty in penetrating the cornea. To improve its ocular delivery, a novel dissolvable MN system (DMN-FNL-IC) using cyclodextrin (CD) inclusion complexes was constructed to enhance FNL properties. By embedding the FNL-CD complex into a PVA/PVP polymer matrix, the system greatly improved the drug’s stability, solubility, and ability to permeate ocular tissue [118]. *In vivo* studies demonstrated that the DMN-FNL-IC system was far more effective than conventional eye drops or ointments. And the HET-CAM (Hen’s Egg Test on the Chorioallantoic Membrane) assay confirmed this system was non-irritated and non-invasive.

Building on this, the researchers used HA and PLA to fabricate MNs. By carefully adjusting the ratio of PLA, the dissolution rate and penetration ability can be fine-tuned. This optimization enabled more rapid penetration of corneal epithelial layer. In animal models, the MN system demonstrated superior bioavailability and therapeutic efficacy compared to traditional eye drops, and its performance was comparable to corneal stromal injection therapy. Impressively, the corneal epithelial defects caused by MN insertion healed completely within 12 h, demonstrating the minimally invasive nature on MN systems.

The researchers also designed a sophisticated two-layer MN system. Its core layer contained eugenol, Tween 80, PEG400 and water, while the shell layer was composed of a chitosan-PVA complex. This smart bilayer structure significantly boosted both the penetration and antifungal power of FNL. This system proved to be remarkable stable, as it retained its strong antifungal efficacy after 3-month storage at both refrigerated (4 °C) and room temperature (RT, 25 °C) conditions [119]. Taken together, these findings indicate that MN technology represents an effective, minimally invasive, and well-tolerated approach for managing fungal keratitis.

#### 3.3.3 Parasitic keratitis

Echinococcus ameba keratitis is a severe infectious disease of the cornea caused by protozoa of the genus *Echinococcus*, and typically affects individuals who are exposed to contaminated water sources or who use contaminated contact lenses [120]. Owing to the drug resistance of *Echinococcus* amoeba, the effectiveness of conventional eye drop treatments is limited, and frequent administration can result in corneal damage or even blindness. For the first time, Ryu’s research team developed a detachable hybrid MN (d-MNP) system for intracorneal injection, enabling sustained drug release. This system combines a degradable (PLGA) tip loaded with the broad-spectrum antimicrobial agent polyhexamethylene biguanide (PHMB) and a nondegradable carrier substrate. By precisely tuning the tip size (∼40 µm) and injection parameters, sustained drug release within the corneal stroma was achieved for over 9 d [121]. In a mouse model of *Echinococcus* amoebic keratitis, the D-MNP system significantly reduced corneal opacity, effectively controlled infection progression, and demonstrated excellent clinical translational potential because of its minimally invasive nature and favorable biocompatibility. Subsequently, the team further optimized the MN system, developing a biodegradable and detachable version to replace traditional, frequent eye drop treatments (Fig. 7C). Various MN tips with different drug concentrations were prepared, with the “high concentration” tip—releasing 12.5 ng PHMB within 3 d—being selected for *in vivo* experiments. In a mouse model of keratitis, a single MN injection replaced the initial 3 d of ophthalmic solution treatment, followed by supplemental eye drop administration for the next 3 d. The combined regimen was found to be as effective as the traditional method of using eye drops for 6 d consecutively, demonstrating the efficacy and potential of MNs in reducing the frequency of treatment and improving patient compliance [42].

In addition, MN-based technology offers significant potential for corneal vaccine delivery, improving treatments for keratitis and related diseases. For example, MN-mediated delivery of antigens such as tetanus toxoid elicited 18- to 30-fold higher antibody responses than intramuscular injection [122]. This remarkable immune enhancement is attributed to several synergistic mechanisms, including the corneal stroma acting as an antigenic “reservoir,” the activation of antigen-presenting cells (APCs), the upregulation of MHC class II molecules and co-stimulatory molecules (CD80/CD86), and lymphatic neovascularization. These findings suggest the considerable potential of MN-based strategies not only for infectious keratitis, but also for immune-mediated and trauma-induced forms of the disease. By enabling rapid, targeted drug delivery and active modulation of the local immune response, this technology provides an effective way for treating corneal inflammation.

To date, research on MNs in the field of virology has primarily been confined to skin diseases or related animal models, such as the use of MNs to track the mechanism of herpes simplex virus type 1 (HSV-1) spread from the skin to the central nervous system. Yet we believe that MNs could also offer a promising new strategy for understanding and treating viral keratitis.

### 3.4 Corneal neovascularization

Corneal neovascularization (CNV) is a sight-threatening condition characterized by the abnormal growth of blood vessels from the limbus into the central cornea. It typically arises in response to corneal hypoxia, inflammation or trauma. CNV can be categorized into three types based on its etiology: inflammation-related CNV, hypoxia-induced CNV and traumatic neovascularization [123]. Current clinical treatments for CNV include anti-VEGF agents, glucocorticoids and laser photocoagulation [124]. However, long-term use of anti-VEGF therapy may lead to drug resistance, while glucocorticoids can cause side effects like elevated IOP and cataract formation. Laser treatment, on the other hand, raises the risk of corneal scarring.

In recent years, wearable corneal MNP have been developed to treat CNV. This system features a water-soluble MN tip attached to a transformable backing layer. In MN tip, antimicrobial nanoparticles (LHAg NPs) were encapsulated, designed to quickly tackle bacterial infection. The backing layer carried epidermal growth factor (EGF) to support corneal tissue repair [125]. Upon application, the tip dissolves immediately, releasing LHAg NPs into the corneal stroma directly. At the same time, the backing layer transforms into a transparent hydrogel that adheres comfortably to the eye, serving as a sustained-release depot for EGF (Fig. 7E). This dual-action system not only fights infection but also creates a favorable microenvironment for healing. *In vitro* studies demonstrated that LHAg NPs exhibited significant antibacterial activity against *S. aureus* and *Streptococcus aureus*, effectively disrupting their biofilms. Beyond disinfection, they also appeared to promote corneal endothelial cell migration and proliferation, accelerating tissue regeneration process. *In vivo* results showed that the MNP significantly reduced corneal healing time compared to traditional eye drops, with sustained EGF release lasting over 8 h. Additionally, it reduced the expression of inflammatory markers (TNF-α, IL-6) and angiogenic factors (VEGF), effectively inhibiting neovascularization. This MNP provides a safe and effective strategy for treating CNV through its dual antimicrobial and pro-repair mechanisms, offering substantial clinical potential.

In another study, Kim et al. [126] developed a coated MN with a tip length of 400 µm that was loaded with the antiangiogenic drug bevacizumab and designed for precise drug delivery to the corneal stroma. In a rabbit model of CNV, drug release was completed only 1 min after MN insertion. The results revealed a 44% reduction in neovascularization by 18 d with only 4.4 µg bevacizumab, representing significantly greater efficacy than topical eye drops (52,500 µg, 6% reduction) and subconjunctival injections (2500 µg, 29% reduction). Histological analysis confirmed that microneedling did not affect corneal transparency and demonstrated good biocompatibility, providing a safe and reliable method for CNV treatment using minimal drug amounts. Using a spring-loaded applicator, Song et al. [19] developed a transfer-molded single MN (MNP) for precise drug delivery to the corneal stroma using a spring-loaded applicator. The MN, with a tip height of 140 µm, avoids penetrating the entire cornea, thus reducing the risk of injury. The MN surface, coated with sunitinib, was shown to significantly inhibit CNV in mice by inhibiting VEGF receptor activity, outperforming conventional injection methods. Than et al. [127] further advanced the technology by developing self-implantable double-layered MNs (DL-MNs), consisting of an HA core and a cross-linked methacrylic acid-HA shell. This double-layered structure achieved a biphasic drug release. The outer layer of the MNs was loaded with the anti-VEGF monoclonal antibody DC101, while the inner layer contained the anti-inflammatory drug diclofenac. This dual-layer system significantly reduced the area of neovascularization by approximately 90%, as diclofenac rapidly inhibited the inflammatory response, whereas DC101 continuously blocked the VEGF signaling pathway. These studies highlight the potential of MNs as an innovative, efficient, and precise therapeutic approach for treating CNV.

### 3.5 Glaucoma

Glaucoma is a chronic and progressive ocular disease primarily characterized by elevated IOP or inadequate blood supply to the optic nerve. It leads to gradual thinning of the optic nerve fiber layer and visual field defects, primarily due to impaired circulation of the intraocular aqueous humor [128]. Based on etiology, glaucoma can be classified as primary open-angle glaucoma, closed-angle glaucoma, or secondary glaucoma [129]. Current clinical treatments for glaucoma include IOP-lowering medications, such as β-blockers and prostaglandin analogues, laser therapies, and surgical interventions, including trabeculectomy [3]. However, the long-term use of IOP-lowering medications is associated with side effects such as ocular irritation and conjunctival congestion. Laser therapy may lose efficacy over time and can induce iris inflammation, whereas surgical interventions carry risks such as postoperative infection and IOP fluctuations [130]. Accordingly, the development of safer, longer-lasting treatment strategies remains a major focus of current research.

In recent years, MNs have emerged as a precise platform for targeted glaucoma therapy. By delivering therapeutics directly into the supraciliary space, this technology significantly cuts the required drug dose, boosts bioavailability, and minimizes systemic side effects. For example, Kim et al. [27] introduced a novel method. They used ultra-fine 33-gauge hollow MNs to inject antiglaucoma drugs, sulprostone and brimonidine, directly into the supraciliary space of rabbit models. After treatment, a dose-dependent reduction in IOP was observed. Impressively, the supraciliary MN delivery used approximately 100 times less drug than conventional eye drops. Safety profiles were also encouraging, although the injections caused a temporary IOP rise similar to vitreous injections; no major adverse effects were observed.

Similarly, Chiang et al. [131] developed a long-acting system using brimonidine-loaded PLA microspheres, delivered via MNs into the same supraciliary region. These microspheres steadily released the drug, maintaining reduced IOP for over a month. Moreover, the researchers also optimized the microsphere formulation by removing low molecular weight acidic impurities, which enhanced drug encapsulation and minimized initial burst release. In rabbits treated with microspheres carrying 0.9 mg brimonidine, IOP was suppressed significantly for more than 30 d. Histological analysis revealed a mild foreign body reaction in the supraciliary space, but no serious adverse effects occurred, and all animals behaved normally. In sum, this sustained-release platform not only extends dosing intervals but also supports better patient adherence.

MNs can also lower IOP by enabling the delivery of drugs or biomaterials directly into the SCS. For instance, a drug-free MN was developed to inject HA hydrogel into the SCS. After injection, the hydrogel crosslinked into a supportive gel structure that physically dilated the space, creating more channels for aqueous humor to drain. This mechanical intervention alone led to a significant and sustained drop in IOP [132]. In a rabbit model, the optimized cross-linked HA formulation (HA-XL) managed to lower IOP without causing any major complications for 4 months, showing great potential as long-acting therapy.

Similarly, Chiang et al. [133] developed a PEG-based hydrogel implant designed for the same target (Fig. 7F). This implant also achieved long-term, and potentially permanent, IOP reduction through physical dilation. *In vitro* experiments demonstrated that SCS dilation could be precisely controlled by adjusting the implant length, and the PEG hydrogel showed good biocompatibility. Both studies confirmed that MN techniques can effectively reduce IOP by physically dilating the SCS, offering a minimally invasive, long-term solution for glaucoma management. This method avoids the challenges associated with traditional medications and high-risk surgical treatments, making it a promising approach for clinical use.

### 3.6 Keratoconus

Keratoconus is a progressive corneal degenerative disease characterized by thinning of the central or paracentral cornea, leading to a conical protrusion [134]. Corneal cross-linking (CXL) is currently an effective treatment for keratoconus that promotes the cross-linking of corneal collagen fibers through the use of riboflavin, which absorbs UV energy, thereby increasing the mechanical strength of the cornea [135]. However, traditional CXL techniques face several significant challenges. The epithelial-off (Epi-off) method, which promotes riboflavin penetration by removing the corneal epithelium, leads to corneal epithelial defects, increasing the risk of postoperative edema and infection and prolonging recovery. Conversely, the epithelial-on (Epi-on) method avoids epithelial damage but suffers from insufficient riboflavin penetration because of the barrier effect of the corneal epithelium, thereby compromising the cross-linking efficacy [136,137].

To address this limitation, Hu et al. [138] developed an innovative responsive porous MN system consisting of a porous gelatin tip, a photothermally responsive PNIPAM/GO hydrogel intermediate layer, and a riboflavin-loaded gelatin backing layer. Under NIR light irradiation, the PNIPAM/GO hydrogel shrinks, facilitating the release of riboflavin into the corneal stroma, thereby enabling highly efficient and minimally invasive CXL therapy (Fig. 7G). *In vitro* experiments demonstrated that this system significantly enhanced the efficiency of riboflavin release under NIR stimulation, promoted the cross-linking of corneal collagen fibers, and improved the biomechanical properties of the cornea. Compared to traditional Epi-on and Epi-off techniques, corneas treated with MNs regained greater transparency, healed faster after surgery, and showed more tightly organized collagen fibers. The system also proved to be highly biocompatible. The corneal epithelium healed completely within just 24 h, with no signs of significant cell death or other complications. By using MNs to deliver riboflavin directly into the stroma, this technique avoids the need to remove the corneal epithelium entirely.

Yang et al. [23] explored MNs with different shapes to deliver riboflavin-HA (RF-HA) solution into the corneal stroma. They are circular, semicircular, triangular, and butterfly-shaped MNs. These MNs gently penetrate the corneal epithelium to a depth of approximately 25 µm. Once insertion, the drug was rapidly released through micropores without damaging the stromal layer. To further enhance riboflavin penetration, an additional RF solution is applied following MN treatment. Under ultraviolet A (UVA) irradiation, that initiate cross-linking between corneal collagen fibers, significantly improving the biomechanical integrity of the tissues. This technique performed comparably to conventional de-epithelialized cross-linking (CXL) in terms of enzymatic degradation resistance and biomechanical strength. More notably, it also allowed faster recovery of corneal epithelium and restoration of thickness. Overall, this novel approach improved both therapeutic efficacy and patient recovery in keratoconus treatment.

## 4 Application of MNs in the treatment of posterior segment eye diseases

Treating posterior segment diseases, such as AMD, DR, and RVO, is challenging in clinical practice. These conditions involve pathological changes in delicate structures including retina, choroid, or vitreous. While conventional IVT can deliver medications directly to these areas, it is highly invasive and associated with risks of endophthalmitis and vitreous hemorrhage. Understandably, many patients are struggled with the anxiety and burden of repeated injections, which may affect treatment consistency. In other respects, the eye’s internal barriers, particularly BRB, are highly effective at keeping foreign substances out. This natural defense prevents most externally applied drugs from reaching the therapeutic levels at the target. As a result, conventional topical therapeutic methods like eye drops seldom achieve sufficient concentration in the posterior segment to be effective, leaving a critical gap in non-invasive treatment options.

By gently penetrating the sclera or conjunctiva, MNs provide a direct and minimally invasive pathway to the posterior segment. MNs can bypass the BRB and deliver drugs precisely to target tissues, thereby significantly increasing drug bioavailability. More importantly, this technique avoids the trauma and complications associated with intravitreal injections. The less invasive inserting procedure also carries a lower risk of infections and allows for quicker recovery. To make MNs smart devices, MNs can be engineered with slow-release systems to extend the duration of drug action, thereby reducing the frequency of administration. This not only helps maintain effective drug levels over time but also makes it easier for patients to stick with their therapy. These advantages provide a novel technical approach for treating posterior segment diseases and offer an important theoretical and practical foundation for further research and development in this area. Table 4 summarizes recent advances of using MNs to treat posterior segment diseases.

**Table 4:** Summary of recent advances in ocular posterior segment diseases treatment by MNs.

Disease model	Modeling method	MN type and characteristics (Tip shape, Array, Length)	Animal model/ Application site	Functions	Effectiveness	Ref.
Wet AMD	Untreated	Hollow MNs, single, 750 µm	New Zealand male white rabbits/SCS	Precise targeting, minimally invasive	Drug spreads rapidly and covers a large area at the back of the eye, and shows good tolerability	[142]
Dry AMD	NaIO_3_-induced retinal degeneration	Dissolving MNs Conical, 5 × 8 grid, 650 µm	New Zealand white rabbits/Sclera	Efficient penetration, precise targeting, minimally invasive, painless	Significant reduction in pro-inflammatory cytokines and increased antioxidant enzyme levels.	[147]
Acute posterior segment inflammation model	Vitreous injection of lipopolysaccharide (LPS)	Hollow MNs, single, 850 µm	Sus scrofa domesticus/SCS	Efficient penetration, precise targeting, minimally invasive	0.2 mg TA microneedle injected into the SCS was comparable to 2.0 mg intravitreal TA in reducing acute posterior segment inflammation.	[149]
Uveitis	Untreated	Biodegradable MNs Conical, 8 × 8, 800 µm	Porcine eye as an *ex vivo* experimental material/SCS	Efficient penetration, precise targeting, minimally invasive, painless	Retention of the microneedle-delivered drug in the sclera was significantly higher than that of the eye drop formulation (*P* < 0.001), and a sustained release was achieved for 7 d.	[150]
Macular edema due to non-infectious posterior uveitis	Untreated	Hollow MNs, single, 900 µm	Human patients/SCS	Efficient penetration, precise targeting, minimally invasive, painless	Significantly reduced CMT, improved best-corrected visual acuity, and controlled inflammation.	[151]
Non-infectious uveitis	Untreated	Hollow MNs (SCS Microinjector™), single, 900 or 1100 µm	Human patients/SCS	Efficient penetration, precise targeting, minimally invasive, painless	Drug diffuses posteriorly and peripherally in the posterior segment, preferentially targeting affected tissues.	[153]
PIOL	Untreated	Biodegradable MNs Tip at a 30° angle, 3 × 3, 2 mm	New Zealand white rabbit/Deep scleral pocket	Precise targeting, minimally invasive	Deliver drug to the choroid and retina without significant side effects.	[159]
High myopia	Modeling the pathology of high myopia by selecting rabbits with specific eye axis lengths and scleral characteristics	Soluble MNs Conical, 400 µm	New Zealand white rabbit/Sclera at the back of eye	Efficient penetration, precise targeting, minimally invasive	Effectively enhance scleral stability and reduce scleral relaxation caused by high myopia.	[162]
CRVO	Untreated	Hollow MNs Angled tip, single	Human patients/Puncture of retinal vein	Efficient penetration, precise targeting, minimally invasive	Within 24 weeks of surgery, 9 patients had a mean improvement in visual acuity of more than 15 letters and a mean reduction in CMT of 271.1 µm.	[168]
CRVO	Untreated	Hollow MNs, Single	Human patients/Puncture of retinal vein	Efficient penetration, minimally invasive	Significantly better visual acuity and lower mean logMAR were seen in 8 of 16 eyes (50%).	[26]
RD	Porcine eyes were stored at −20 °C and thawed at RT to artificially induce RD.	Hollow MNs 15° taper at the tip of needle, single, overall needle length 5 mm	Enucleated pig eye/RD area	Efficient penetration, precise targeting, and minimally invasive	Within 5 min, 1000 µl subretinal fluid was drained, and the RD was completely reattached.	[170]
/	Untreated	Hollow MNs, single, 700 µm	Macaca mulatta/SCI and SRI	Efficient penetration, precise targeting, minimally invasive	Demonstrated the safety and efficacy of AAV gene therapy by producing localized, strong gene expression.	[60]
Retinal damage and oxidative stress-related diseases induced by iron overload	Untreated	Soluble MNs, 3 × 3, 700 µm	Enucleated pig eye/Sclera	Efficient penetration, precise targeting, minimally invasive	After insertion into the porcine sclera, drug deposition efficiency reached 64% within 5 min, creating a localized high concentration distribution in the retina to more effectively reduce iron overload and oxidative stress.	[24]

### 4.1 Age-related macular degeneration

Age-related macular degeneration (AMD) is a chronic, progressive ocular degenerative disease characterized by dysfunction of the RPE and choroidal vasculopathy, leading to structural damage to the macula and functional loss [139]. Based on its pathological features, AMD can be classified into two types: dry (non-neovascular) and wet (neovascular). Current clinical treatments include anti-VEGF therapies, photodynamic therapy (PDT), and nutritional supplementation [140]. However, the long-term use of anti-VEGF agents is associated with drug tolerance and an increased risk of ocular infections. PDT offers limited efficacy and is associated with a risk of retinal damage, and the efficacy of nutritional supplements in slowing disease progression remains uncertain. Consequently, the development of safer and longer-lasting therapeutic strategies has become a primary focus of ongoing research.

Wet AMD, characterized by the abnormal growth and leakage of CNV, often leads to subretinal hemorrhage, edema, and fibrosis, severely compromising central vision [141]. While the IVT of anti-VEGF agents remains the standard of care, the need for frequent administration imposes a significant burden on patients and increases the risk of procedure-related complications. To address these limitations, Jung et al. [142] developed an MN-based *in situ*-forming hydrogel system for the targeted delivery of bevacizumab into the SCS. Utilizing 750-µm-long MNs, this minimally invasive approach enables the precise delivery of a low-viscosity hydrogel that cross-links *in situ* to form a stabilized gel depot, providing sustained drug release for up to 6 months. By sequestering VEGF and preventing its receptor binding, bevacizumab reduces subretinal fluid accumulation and hemorrhage, effectively inhibiting neovascularization and leakage. *In vivo* studies demonstrated excellent ocular tolerability and significant therapeutic efficacy, highlighting the potential of the system to serve as a long-acting, safe, and effective treatment for wet AMD.

**Fig. 8:**
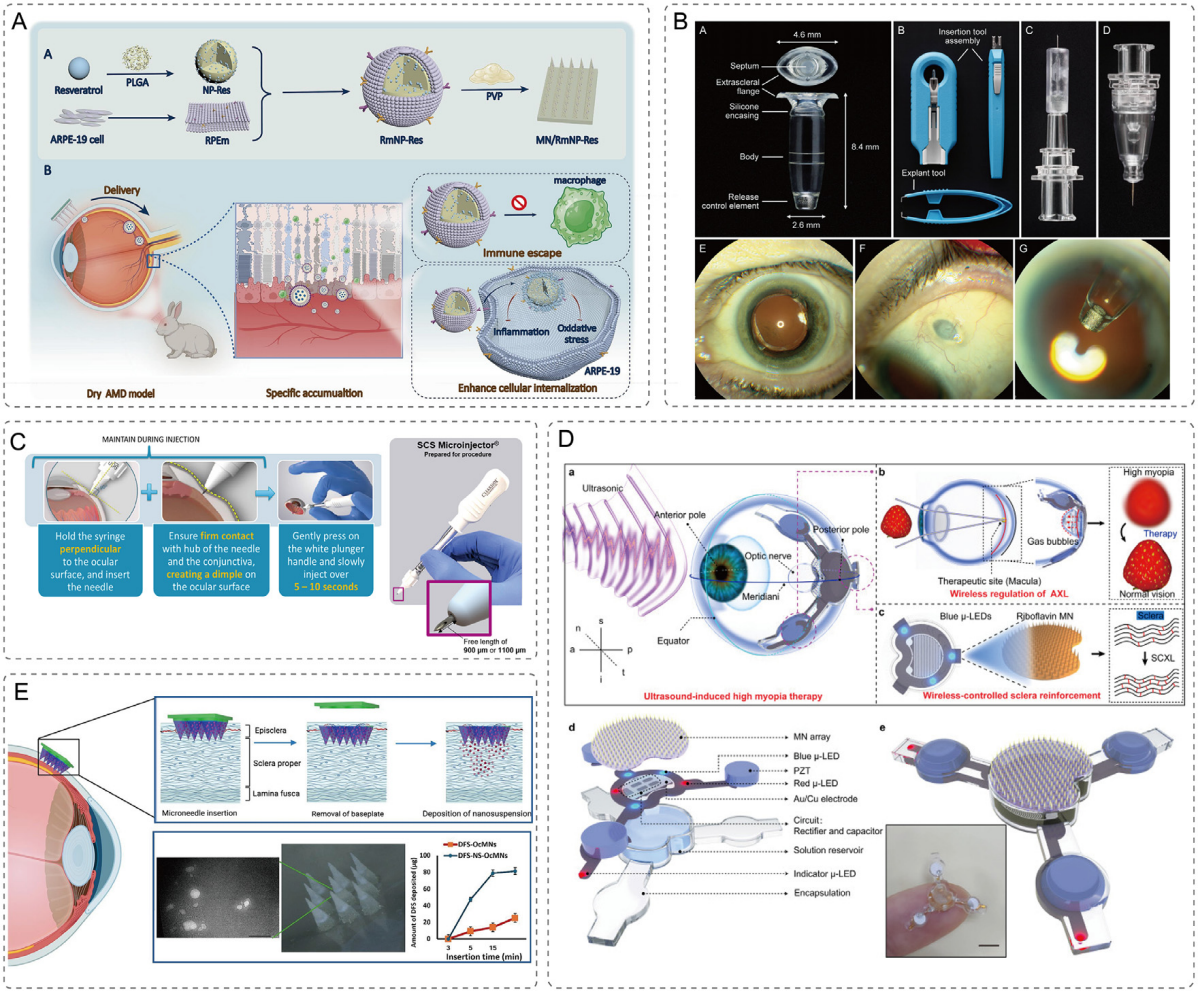
Recent advances of MNs for posterior segment ocular diseases. (A) MN-enabled targeted therapy for dry AMD Reprint with permission from [147]. Copyright© 2025 Elsevier B.V. ; (B) Port Delivery System for retinal delivery. Reprint with permission from [143]. Copyright© 2022 The Author(s).; (C) SCS Microinjector® for subchoroidal injection. Reprint with permission from [153]. Copyright© 2020 The Authors; (D) Multifunctional MNP for high-myopia therapy. Reprint with permission from [162]. Copyright© 2024 The Author(s); (E) Dissolving MN for retinal disease therapy. Reprint with permission from [24]. Copyright© 2024 The Authors.

The U.S. Food and Drug Administration (FDA) has approved a refillable intraocular microreservoir implant named the Port Delivery System (PDS, marketed as Susvimo^Ⓡ^) for long-term anti-VEGF therapy in patients with wet AMD. Through minimally invasive surgery, a transparent drug reservoir measuring 4.6 mm × 8.4 mm is secured 3.5–4 mm behind the corneoscleral limbus. Prefilled with 20 µl of ranibizumab at a concentration of 100 mg/ml, the implant exhibits zero-order drug release sustained for more than 6 months via a porous titanium release control element (RCE) on its surface. A matching 34-G dual-lumen refill needle allows for one-time replacement of up to 95% of the drug solution, facilitating *in situ* refilling without the need for repeated intravitreal injections (Fig. 8B) [143]. The phase II Ladder study demonstrated that 80% of patients in the 100 mg/ml group did not require supplemental dosing for 6 months, with visual and anatomical outcomes comparable to those achieved with monthly injections of 0.5 mg ranibizumab. Transient postoperative vision decline was quickly reversed and was maintained long term [144]. In summary, the PDS significantly reduces the treatment burden associated with long-term anti-VEGF therapy and offers a novel approach to managing AMD.

Expanding beyond VEGF-dependent pathways, Galvin et al. [145] developed an HA-based MN system for the delivery of quininib, a leukotriene receptor antagonist with antiangiogenic effects independent of VEGF signaling. *In vitro* release studies revealed an initial burst release of quininib from the HA MNs, followed by sustained release modulated by hyaluronidase activity. *In vivo* experiments demonstrated that the quininib-loaded HA MNs significantly inhibited retinal angiogenesis in a zebrafish model and reduced retinal vascular leakage in a rat model, with therapeutic effects lasting for at least 1 month, suggesting strong potential for the long-term treatment of AMD.

Additionally, Gade et al. [146] developed a thermoresponsive CS-grafted poly(N-isopropylacrylamide) (Cs-g-PNIPAAm) hydrogel for the delivery of sunitinib, a multitargeted tyrosine kinase inhibitor that blocks VEGF receptor (VEGFR) and platelet-derived growth factor receptor (PDGFR) signaling. The hydrogel was injected into the sclera using a 27-G needle, forming a stable drug reservoir at physiological temperature for sustained release. *In vitro* studies showed that a 20 µl injection of 30% Cs-g-PNIPAAm hydrogel enabled sustained sunitinib release at approximately 10 µg/d for 28 d. In a rat CNV model, abnormal blood vessel growth was strongly suppressed after treatment, demonstrating potent anti-angiogenic properties of this system. Beyond its efficacy, the optimized hydrogel proved to be highly biocompatible with ARPE-19 cells. In the HET-CAM test, no signs of irritation were observed, indicating that this hybrid MN and hydrogel delivery system has potential for safe clinical AMD treatment.

Dry AMD constitutes 80% to 90% of all AMD cases. This condition is primarily driven by retinal damage from oxidative stress and inflammation. Given the lack of effective treatments, Liu et al. [147] developed a bio-inspired nanoparticle delivery system utilizing dissolving MNs. RPE cell membrane coated PLGA nanoparticles was used to encapsulate resveratrol, a compound known for its potent antioxidant properties (Fig. 8A). Then the nanoparticles were loaded in MNs. After insertion in sclera on an *ex vivo* porcine eye model, this MN system dissolved completely within 5 min at 37 °C. This rapid dissolution facilitates the intraocular release of the bio-membrane coated nanoparticles. After release, the homologous targeting of RPE membrane would lead the way for the nanoparticles to accumulate in retinal tissues, thereby achieving precise delivery of the therapeutics. In animal studies, this system successfully delivered and retained resveratrol in retinal tissues, exhibiting excellent antioxidant and anti-inflammatory capabilities to improve fundus lesions and reduce RPE degeneration. Importantly, the tiny micropores created by the MNs healed within 1 h without disrupting IOP, showing MNs a safe and effective strategy for AMD therapy.

### 4.2 Uveitis

Uveitis is a leading cause of visual impairment, particularly when inflammation involves the posterior segment of the eye, which presents significant treatment challenges [148]. Traditional therapeutic approaches, including topical or systemic glucocorticosteroids, are limited by poor drug penetration across the blood-retinal barrier, systemic side effects, and the necessity for frequent administration. In recent years, SCS injections have attracted great attention. By injecting therapeutics directly into the SCS, this technique enhances drug availability in the posterior segment. As the medication remains largely confined to this space, it limits exposure to both anterior chamber and the vitreous body, minimizing unwanted side effects.

In the early studies on SCS injection, researchers mainly focused on its feasibility and safety. For example, Gilger et al. [149] used an MN-based system to administer triamcinolone acetonide (TA) into the SCS in a porcine model of acute posterior uveitis.

After treatment, the inflammation was effectively controlled, and no significant adverse effects, such as elevated IOP or drug-induced toxicity, occurred. This work provided foundational evidence that SCS injections could be both safe and effective in large animals, paving the way for future clinical applications.

During preclinical development, Shelley et al. [150] developed a biodegradable MNP based on PLGA. This PLGA MN system was designed to provide sustained release of the anti-inflammatory drug. The MNs were shown to penetrate the sclera uniformly and release the drug at a consistent, controlled rate over 7 d. With an excellent biocompatibility of PLGA, this MNP showed great potential in advancing SCS drug delivery toward clinical applications.

In a key clinical study, Anand et al. [151] conducted a prospective trial to evaluate a single, low-dose (2 mg) SCS injection of tretinoin in patients suffering from macular edema due to noninfectious posterior uveitis. Within 12 weeks of treatment, 9 out of 10 patients showed significant reductions in macular thickness and meaningful improvements in visual acuity. Importantly, no significant elevations in IOP were detected, marking the first clinical validation of both the efficacy and safety of SCS injections for this condition. In 2021, XIPERE® (TA injectable suspension for suprachoroidal use, 4 mg/0.1 ml) has been approved by FDA for the treatment of macular edema associated with noninfectious uveitis. This therapy is administered using the SCS Microinjector® developed by Clearside Biomedical. Using a 30-G MN with either 900 µm or 1100 µm in length, the sclera is penetrated perpendicularly 4–4.5 mm posterior to the limbus to precisely deliver TA into the SCS [152]. The drug then diffuses toward the posterior pole and macular region under the natural ocular pressure gradient, achieving targeted enrichment, compartmentalization, and sustained release (Fig. 8C). Wan et al. [153] compiled baseline data from six trials, including AZALEA, PEACHTREE, and TYBEE, confirming that both 30-G MNs with 900 µm and 1100 µm in length can safely and effectively establish a suprachoroidal drug delivery pathway. Among these, 71% of the injections were completed using only the 900 µm needle, with an 84% physician satisfaction rate and no instances of unintended intravitreal injection. This fully demonstrates the individual adaptability and clinical operability of MN technology across diverse patient populations.

By delivering drugs directly into the SCS, this technology offers a smarter, more targeted approach for treating uveitis. The benefits include improved drug bioavailability in the posterior segment, reduced systemic and ocular side effects, and a lower administration frequency. The progressive development of SCS injections from concept to clinic demonstrates its strong potential for treating diseases in the back of the eye. As clinical research expands and the technology continues to advance, SCS injections are poised to become a vital therapeutic option for uveitis and other posterior segment disorders.

### 4.3 Ocular tumors

Uveal melanoma and Rb are common ocular tumors that happened in the back of the eyes. These cancers are shielded by natural barriers like BRB and the cornea, making them difficult to be accessed by medications. Conventional treatment modalities contain surgical resection, radiation therapy, systemic chemotherapy, and local injections. However, all of them are associated with substantial limitations. Surgical resection is highly invasive and can lead to permanent vision loss. Radiation therapy offers targeted action but carries a high risk of causing radiation retinopathy. Systemic chemotherapy is limited by severe systemic toxicity, including bone marrow suppression, hepatic dysfunction and renal dysfunction. Although local intravitreal injections can increase intraocular drug concentrations, they require repeated procedures, increasing the risk of infection and tumor migration. These challenges highlight the urgent need for safer and more effective therapeutic strategies. Recent studies showed that MN-based systems can efficiently deliver a range of agents, such as chemotherapeutics [154], gene therapies [155], and photosensitizers [156]. Those MN platforms effectively inhibited tumor growth while lowering both systemic and local toxicity. Their high biocompatibility and the ability to release drugs in a controlled manner make them appealing for clinical use. Looking ahead, combining MNs with smart nanomaterials could further enhance the precision, efficiency, and safety of therapies targeting ocular tumors,

Primary intraocular lymphoma (PIOL) is a nonmetastatic malignant lymphoma that affects the vitreous, retina, or choroid. It occurs when malignant B lymphocytes infiltrate these areas and disrupt RPE [157]. The current standard treatment for PIOL involves intravitreal injections of methotrexate. Unfortunately, this method leads to uneven drug concentrations, cumulative toxicity, as well as complications induced by repeated injections [158].

To overcome these limitations, Palakurthi et al. [159] designed a biodegradable MN implant made of PLA, to slowly release methotrexate intraocularly. The implant was fabricated using a combination of solvent casting and micromolding techniques. In a rabbit model, the MN implant was precisely delivered into the deep scleral pocket without inducing any complication, demonstrating both accuracy and minimal tissue disruption. In histopathological analysis, no signs of acute inflammation or infection were found in surrounding ocular tissues. This demonstrated methotrexate-induced toxicity was neglectable. As MN technology offers a less invasive and more reliable option for managing PIOL, it could also serve as an adaptable platform for treating other posterior segment tumors. With favorable safety profile, sustained delivery capabilities, and clear clinical potential, MN technology highlights significant prospects for application in ocular oncology.

### 4.4 Myopia

Myopia, also known as short-sightedness, is a common refractive eye disorder. Its global prevalence is rising rapidly. According to the WHO, by 2050, nearly half of the global population is projected to be affected by myopia, with high myopia (refractive error worse than −5.0 diopters) accounting for approximately 20%, or 911 million people [160]. This alarming trend represents a major global public health challenge. The pathophysiology of myopia primarily involves excessive axial elongation of the eye or increased refractive power of the cornea and lens, resulting in parallel light rays focusing in front of the retina rather than directly upon it. Clinically, this manifests as blurred distance vision while near vision remains relatively clear [161].

Current corrective methods, including spectacles, contact lenses, and refractive surgery, are effective for improving visual acuity but remain inadequate for halting myopia progression. In response to these limitations, Zhong et al. [162] developed an innovative wireless, battery-free ocular alignment patch that integrates a soluble MN array with a light-activated scleral collagen cross-linking (SCXL) system to address the progression of high myopia (Fig. 8D). The MNs, fabricated from a PVP-riboflavin blend and precision-molded using polydimethylsiloxane (PDMS) templates, feature a tapered design to minimize scleral surface penetration while ensuring efficient riboflavin delivery to the target area. Embedded miniature blue LEDs (443 nm) initiate the photochemical activation of riboflavin, generating ROS that induce collagen fiber cross-linking, thereby enhancing scleral biomechanical strength. *In vivo* experiments revealed a 387% increase in the scleral Young’s modulus at 22 d after treatment, indicating effective suppression of the pathological axial elongation. Histological analysis confirmed a MN penetration depth of approximately 84 µm, which was restricted to the scleral layer without damaging adjacent choroidal or retinal tissues, and no adverse effects, such as inflammation or fibrosis, were observed. Furthermore, the incorporation of wireless ultrasound power supply technology eliminated the need for external wired connections, significantly improving treatment safety and usability. This synergistic MN-optical approach overcomes the invasive nature of traditional scleral buckling procedures, providing a precise, minimally invasive, and innovative therapeutic solution for managing high myopia, with substantial potential for clinical translation.

Additionally, to further enhance the diagnostic and therapeutic capabilities for myopia management, MN technology can be integrated with advanced platforms such as biomarker detection systems [163], microfluidic chips [164], and wearable devices [165], paving the way for comprehensive and personalized treatment strategies.

### 4.5 Retinal diseases

Central retinal vein occlusion (CRVO) is a prevalent cause of vision loss among patients with retinal vascular diseases and is characterized by retinal vein obstruction, macular edema and subsequent visual impairment [166]. Central retinal thickness (CMT) serves as a critical indicator for assessing macular edema severity and is closely correlated with visual prognosis [167]. MNs facilitate the direct injection of thrombolytic agents, such as tissue plasminogen activator (tPA), into occluded retinal veins, thereby improving retinal circulation and visual acuity. Kadonosono et al. [168] developed a stainless-steel MN with an outer diameter of 50 µm for retinal vein puncture. In a clinical study, nine patients exhibited a mean improvement in visual acuity exceeding 15 letters and a mean CMT reduction of 271.1 µm within 24 weeks postoperatively. Subsequently, Ishida et al. [26] confirmed these findings, demonstrating significant visual acuity improvement in patients with nonischemic CRVO 6 months after treatment. However, the technique exhibited limited efficacy in ischemic CRVO, indicating the need for further optimization tailored to specific pathological subtypes.

Retinal diseases (RD), an ophthalmologic emergency, is characterized by the separation of the neuroretinal layer from the underlying retinal pigment epithelium, resulting in acute vision loss and visual field defects [169]. Ma et al. [170] developed a curved MN (CMD) with a unique structure (100 µm outer diameter, 80 µm inner diameter, and 0.8 inner/outer diameter ratio) designed for precise subretinal fluid drainage while minimizing tissue trauma. In a porcine eye model, the CMD effectively evacuated 1000 µl of subretinal fluid, restored retinal attachment, and preserved the structural integrity of the retina and choroid, as confirmed by histological analysis. These findings highlight the potential of the CMD as a minimally invasive surgical tool for RD management, reducing procedural complications and expanding the applications of MNs in retinal surgery.

In the field of gene therapy, Yiu et al. [60] explored the use of MNs for suprachoroidal injection to deliver AAV8 vectors. Their study showed that suprachoroidal injection facilitated broad transduction across the peripheral retina, whereas subretinal injection led to more localized gene expression. Compared to conventional vitreous injections, MN-mediated delivery of AAV8 resulted in higher transduction efficiency in both the subretinal space and the SCS. It also elicited a reduced systemic immune response. In non-human primate models, peak transgene expression occurred 1 month after suprachoroidal injection, though expression levels decreased after 2–3 months. These findings highlight the potential of MN-based delivery to enhance the efficacy of retinal gene therapy while reducing immune-related complications.

Iron overload triggers retinal degeneration through oxidative stress and inflammation. Conventional iron chelator therapies are hindered by poor ocular bioavailability and low patient compliance [171]. To overcome these limitations, Faizi et al. [24] developed an MN system loaded with a nanosuspension of the iron chelator deferasirox (Fig. 8E). *In vitro* tests showed that under a constant 3 N force, the MN penetrated porcine scleral tissue to an average depth of 81.23% ± 7.35% of its total length, as measured by optical coherence tomography (OCT). This indicates the system is robust enough to effectively deliver its payload across sclera to retina, therefore improving the bioavailability of iron chelator. The system was also proved biocompatible as ARPE-19 cell viability maintained above 70% during co-incubation. Notably, the researchers found that this MN system deposited nearly five times more drug into scleral tissue than a conventional MN, demonstrating its improved delivery efficiency. These results support MN-based systems as an efficient and patient-friendly approach for treating iron overload-related retinal injury, thereby expanding the potential applications of MN technology in retinal disease therapy.

In summary, MN technology provides a versatile and minimally invasive platform for treating various RDs, such as vascular occlusions, RD, gene therapy-related conditions, and iron overload injury. Its precision targeting, improved delivery efficiency, and favorable safety profile highlight its transformative potential in retinal therapeutics.

## 5 Conclusion

Ophthalmic MN technology has emerged as a highly promising platform for treating ocular diseases, owing to its unique design and targeted drug delivery mechanisms. Unlike conventional ocular formulations, MNs can effectively overcome key physiological barriers in the eye, including the cornea, sclera, and BRB, enabling precise, minimally invasive, and efficient drug administration. This approach significantly improves drug bioavailability while minimizing systemic exposure and related side effects. Recent studies have demonstrated the efficacy of MN-based therapies in managing both anterior segment diseases like keratitis, dry eye, and glaucoma, and posterior segment disorders, such as AMD and DR. Different types of MN designs offer tailored solutions for various ocular indications. Soluble MNs, which gradually degrade to release their payloads, are particularly suited for chronic ocular surface diseases requiring sustained drug delivery. Hollow MNs facilitate the controlled infusion of large molecules, providing a novel approach to penetrate the blood-retinal barrier. Coating the surface of MNs with therapeutics allows rapid drug release and is advantageous for managing acute inflammatory conditions. Moreover, by adjusting parameters such as tip length, needle density, and substrate geometry, MNs can be customized to meet diverse therapeutic requirements, offering a platform for personalized ophthalmic treatments.

MN-mediated gene delivery is regarded as a highly promising minimally invasive treatment strategy in the field of ophthalmology. Although only a few studies have reported cases in which MN are combined with AAV vectors, existing research has demonstrated that subretinal injection of a recombinant adeno-associated virus type 2 vector carrying the soluble VEGF receptor-1 (sFLT-1) gene (rAAV2-sFLT-1) is safe and well tolerated in patients with wet AMD. This system can reduce the long-term need for anti-VEGF injections [172]. Furthermore, IVT of HIV-1-derived VSV-G pseudotyped lentivirus (LV) can be used to successfully deliver the CRISPR/Cas9 system along with guide RNA (gRNA) targeting myocilin (MYOC) for exploratory glaucoma treatment [173]. These studies not only validate the therapeutic efficacy of multiple viral vectors in the eye but also provide an important theoretical and experimental foundation for the further development of gene delivery systems that combine MNs with non-AAV viral vectors.

Despite the promising potential, several challenges hinder the clinical translation and widespread adoption of ocular MN technologies. The MN manufacturing process is complex and requires precise control over dimensions, material properties, and fabrication techniques to ensure safety and therapeutic efficacy. In large-scale production, the height, curvature, and tip sharpness of MNs must be maintained at micron-level precision. Yet, high-throughput injection molding, hot embossing, or 3D printing frequently introduce burrs, blunted tips, or array irregularities, causing marked variability in penetration efficiency. These place extreme demands on equipment stability and process windows [174,175]. Moreover, ocular formulations must be sterile. Conventional sterilization methods such as high-temperature treatment or γ-irradiation often inactivate the loaded drugs or destroy the MN structure. Consequently, developing a sterilization route that is gentle to sensitive therapeutics while remaining compliant with GMP standards remains a critical challenge. Issues related to biocompatibility and biodegradability remain critical, necessitating further optimization to minimize immune responses and tissue damage. For example, non-metallic inorganic materials such as silicon and glass are frequently employed in solid MNs. These materials offer excellent hardness but suffer from limited toughness. As a result, extremely fine tips made from these materials may break during insertion into the sclera, leaving behind tiny, non-degradable fragments in the eye. These residual pieces could cause ongoing tissue irritation or even lead to chronic inflammation. Certain metallic needle materials, such as titanium (Ti), have been reported to exhibit potential cytotoxicity as well [184].

Additionally, regulatory and market-related barriers also pose significant hurdles. As medical devices, MNs are subject to rigorous regulatory oversight, with variations in classification and approval processes across countries. For instance, in China, MN products must undergo comprehensive sterility validation, risk assessment, and efficacy evaluations. Production and business licenses must be obtained prior to market entry. From an industrial perspective, challenges such as limited drug loading capacity, scaling difficulties, and compliance with good manufacturing practice (GMP) standards complicate mass production. Moreover, patient acceptance of MN-based ocular treatments remains relatively low, partly because of concerns regarding safety and unfamiliarity with the technology. Compounding these issues is the absence of unified quality standards and regulatory frameworks, which further limit clinical application and market adoption.

Ophthalmic MN technology is expected to evolve through multidimensional innovations. Advances in materials science, nanotechnology, and biotechnology will facilitate the development of intelligent, stimuli-responsive and highly biocompatible MNs, thereby increasing therapeutic precision and safety. The integration of microstructures with smart materials will enable more accurate spatiotemporal control of drug delivery in response to specific microenvironments of ocular tissues. 3D printing and microfluidic technologies will enable precise control over MN geometries, optimizing their design for specific ocular tissues, such as the cornea and retina. Furthermore, artificial intelligence-assisted MN design holds promise for achieving personalized, patient-specific treatments, paving the way for individualized “eye-customized” therapeutic solutions. These technological advancements are expected to drive the clinical translation of MN technology, offering safer, more effective, and patient-centered treatments for ocular diseases.

## CRediT authorship contribution statement

**Jing Li:** Writing – original draft, Investigation, Conceptualization. **Zi Yan:** Validation, Investigation, Formal analysis, Data curation. **Dengxuan Mao:** Investigation, Data curation. **Xiumei Liu:** Investigation, Data curation. **Peixin Lee:** Investigation. **Yaqi Lyu:** Writing – review & editing, Validation, Funding acquisition, Conceptualization. **Nianping Feng:** Writing – review & editing, Supervision, Resources, Funding acquisition, Conceptualization.

## Conflicts of interest

The authors report no conflicts of interest. The authors alone are responsible for the content and writing of this article.
